# Exploring the Structures and Functions of Macromolecular SLX4-Nuclease Complexes in Genome Stability

**DOI:** 10.3389/fgene.2021.784167

**Published:** 2021-11-04

**Authors:** Brandon J. Payliss, Ayushi Patel, Anneka C. Sheppard, Haley D. M. Wyatt

**Affiliations:** ^1^ Department of Biochemistry, Temerty Faculty of Medicine, University of Toronto, Toronto, ON, Canada; ^2^ Canada Research Chairs Program, Temerty Faculty of Medicine, University of Toronto, Toronto, ON, Canada

**Keywords:** SLX1-SLX4, MUS81-EME1, XPF-ERCC1, SLX4IP, structure-selective endonuclease, homologous recombination (HR), genome stability

## Abstract

All organisms depend on the ability of cells to accurately duplicate and segregate DNA into progeny. However, DNA is frequently damaged by factors in the environment and from within cells. One of the most dangerous lesions is a DNA double-strand break. Unrepaired breaks are a major driving force for genome instability. Cells contain sophisticated DNA repair networks to counteract the harmful effects of genotoxic agents, thus safeguarding genome integrity. Homologous recombination is a high-fidelity, template-dependent DNA repair pathway essential for the accurate repair of DNA nicks, gaps and double-strand breaks. Accurate homologous recombination depends on the ability of cells to remove branched DNA structures that form during repair, which is achieved through the opposing actions of helicases and structure-selective endonucleases. This review focuses on a structure-selective endonuclease called SLX1-SLX4 and the macromolecular endonuclease complexes that assemble on the SLX4 scaffold. First, we discuss recent developments that illuminate the structure and biochemical properties of this somewhat atypical structure-selective endonuclease. We then summarize the multifaceted roles that are fulfilled by human SLX1-SLX4 and its associated endonucleases in homologous recombination and genome stability. Finally, we discuss recent work on SLX4-binding proteins that may represent integral components of these macromolecular nuclease complexes, emphasizing the structure and function of a protein called SLX4IP.

## 1 Introduction

Slx1 and Slx4 (synthetic lethal of unknown function) were discovered in *Saccharomyces cerevisiae* in a synthetic lethality screen to identify proteins required for the viability of cells lacking the Sgs1 helicase (Mullen et al., 2001). Sgs1 is a member of the RecQ family of helicases, which have essential roles in DNA replication and homologous recombination because of their ability to unwind DNA secondary structures (Lu and Davis, 2021). The human ortholog is Bloom’s syndrome helicase (BLM), so named because of its deficiency in patients suffering from Bloom’s syndrome (Ababou, 2021). The genetic phenotypes of *slx1*Δ and *slx4*Δ cells, including genotoxin sensitivity, colony morphology and sporulation efficiency, suggested that Slx1 and Slx4 functioned together in response to DNA damage. Indeed, coimmunoprecipitation experiments confirmed an interaction between the endogenous proteins (Mullen et al., 2001). Soon after, *Schizosaccharomyces pombe* Slx1 and Slx4 were shown to form a stable protein complex that was essential for viability in the absence of the Rqh1 helicase (Coulon et al., 2004). The genetic relationship with Sgs1/Rqhl immediately suggested that Slx1-Slx4 could have an essential role in removing the branched DNA structures, also known as joint molecules, that form during replication and homologous recombination.

**FIGURE 1:**
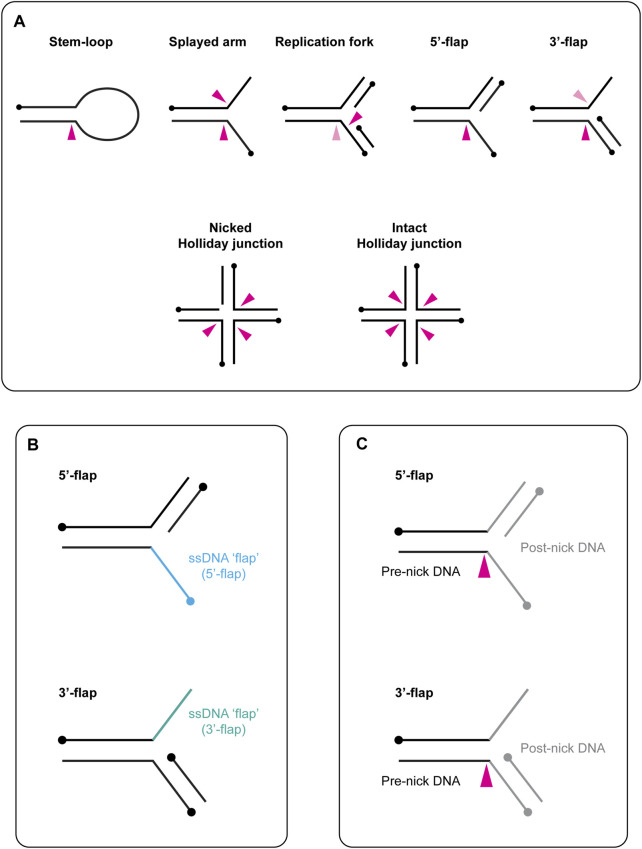
**(A)** Schematic representation of branched DNA structures that are cleaved *in vitro* by yeast Slx1-Slx4 and human SLX1-SLX4. Black circles indicate 5′-termini. A pink arrow indicates the approximate sites of cleavage within each DNA structure, with a dark pink arrow representing the predominate cleavage site and a light pink arrow representing the minor cleavage site. When multiple arrows are shown, each arrow represents one possible cut site. **(B)** Graphical description of flapped DNA structures. The single-stranded DNA (ssDNA) is referred to as the flap and is shown in blue (5′-flap, top) or green (3′-flap, bottom). The name of the DNA substrate reflects the polarity of the flap: the ssDNA runs 3′ to 5′ in the 5′-flap **(top)** and 5′ to 3′ in the 3′-flap **(bottom)**. Circles indicate 5′-termini. **(C)** Schematic representation of the terms pre-nick and post-nick, which are used to distinguish between the two double-stranded segments of DNA that flank the incision site. Black lines represent the double-stranded DNA upstream of the incision site (pre-nick), whereas grey lines represent the double-stranded DNA downstream of the incision site (post-nick). Circles indicate 5′-termini. A pink arrow indicates the approximate sites of cleavage within each DNA structure.

Biochemical studies revealed that yeast Slx1-Slx4, as well the vertebrate counterparts (referred to here as SLX1-SLX4), cleave a variety of branched DNA substrates, including stem-loops and Y-structures (also known as splayed arms), replication forks, 5′- and 3′-flaps, and nicked or intact Holliday junctions (Figure 1A) (Fricke and Brill, 2003; Coulon et al., 2004; Fekairi et al., 2009; Svendsen et al., 2009; Wyatt et al., 2013; Gaur et al., 2015; Wyatt et al., 2017; Gaur et al., 2019). As described in more detail below, Slx1-Slx4/SLX1-SLX4 cleaves the phosphodiester backbone on the 3′-side of the DNA branchpoint. Together with the finding that Slx1-Slx4 does not cleave single-stranded or fully duplexed DNA, these observations explain its classification as a structure-selective endonuclease. Like some other structure-selective endonucleases, including Rad1-Rad10/XPF-ERCC1 and Mus81-Mms4/MUS81-EME1, Slx1-Slx4/SLX1-SLX4 is an obligate heterodimer that contains one catalytic subunit (Slx1/SLX1) and one non-catalytic subunit (Slx4/SLX4); the interaction between these subunits is essential for nuclease activity.

The ability of Slx1-Slx4/SLX1-SLX4 to cleave a wide range of branched DNA structures underpins its critical roles in DNA repair (i.e., homologous recombination and interstrand crosslink repair), the cellular response to replication stress and telomere homeostasis (Guervilly and Gaillard, 2018). The physiological role of SLX4 is underscored by the fact that biallelic mutations in *SLX4* can lead to Fanconi anemia (Kim et al., 2011; Stoepker et al., 2011; Schuster et al., 2013). Fanconi anemia is a rare recessive disorder characterized by congenital abnormalities, progressive bone marrow failure, genome instability and predisposition to cancer (Niraj et al., 2019). Cells derived from these patients are exquisitely sensitive to compounds that cause DNA interstrand crosslinks. Additionally, there are some reports of cancer-predisposition or cancer-associated mutations in *SLX4* (Bakker et al., 2013; Shah et al., 2013; Sousa et al., 2015; Torrezan et al., 2018).

This review aims to synthesize what is known about the structure and functions of SLX1-SLX4. After a brief overview of the evolution of Slx1/SLX1 and Slx4/SLX4 in eukaryotes, we will discuss the structural and biochemical properties of fungal Slx1-Slx4 proteins, for which several high-resolution structures are now available. Here, we assimilate our knowledge on how Slx1-Slx4 recognizes and cleaves branched DNA structures and highlight important questions for the future. After summarizing the conserved functions of Slx1-Slx4/SLX1-SLX4 in homologous recombination, we discuss the multifaceted roles that are fulfilled by human SLX1-SLX4 in genome stability. This section is organized around the structure and functions of human SLX4 and the macromolecular nuclease complexes that assemble on the SLX4 scaffold. Finally, we discuss recent work on SLX4-binding proteins that may represent integral components of these macromolecular nuclease complexes, emphasizing the structure and function of SLX4IP. Throughout this review, we highlight current knowledge gaps and discuss multiple perspectives in areas of uncertainty.

### 1.1 A Note on DNA Substrate Terminology

In this review, we use the term “branchpoint” to refer to a malleable discontinuity in the DNA double helix. Common examples of branchpoints include the junction between single-stranded (ss) and double-stranded (ds) DNA in the stem-loop, the junction in a three-armed structure (e.g., splayed arm, replication fork, 5′-flap) and the core of a four-stranded Holliday junction (Figure 1A). Concerning the nomenclature of flapped DNA structures, the ssDNA is referred to as the “flap,” and the substrate name reflects the polarity of this strand (e.g., the 5′-flap contains an extension of ssDNA at the 5′-end) (Figure 1B). We use the terms “pre-nick” and “post-nick” to distinguish between the two dsDNA segments (or arms) that flank the incision site. Specifically, “pre-nick” refers to dsDNA upstream of the incision site, while “post-nick” refers to dsDNA downstream of the incision site (Figure 1C).

## 2 Evolution of Slx1/SLX1 and Slx4/SLX4 in Eukaryotes

### 2.1 Slx1/SLX1 Belongs to the GIY-YIG Nuclease Superfamily

**FIGURE 2:**
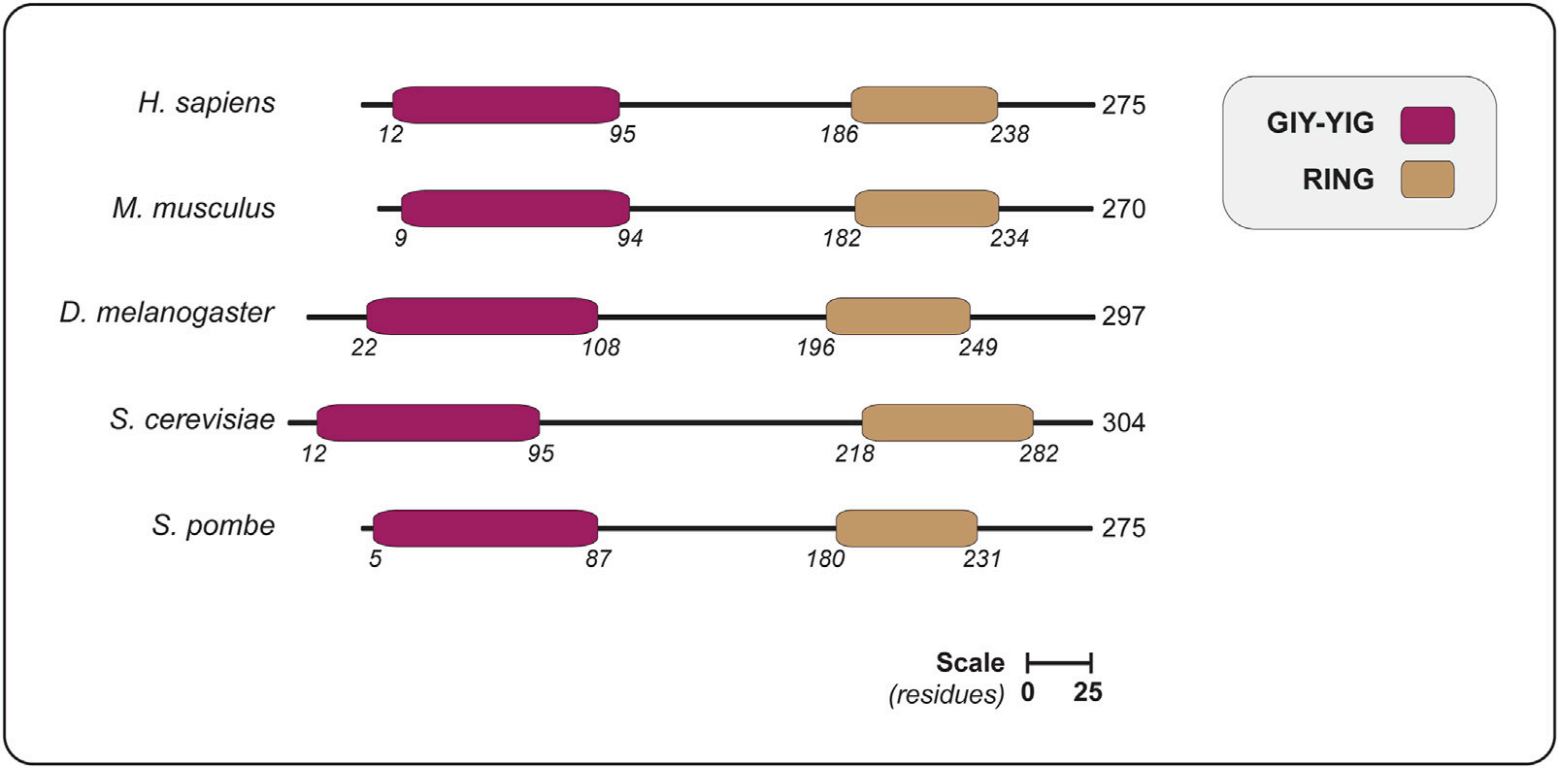
The domain organization of SLX1 proteins from different eukaryotes, showing the positions of the GIY-YIG nuclease domain and RING domain. Domains are colored as indicated in the legend (upper right). The length of each protein is indicated in amino acids, as are the domain boundaries. All the proteins are shown on the same scale (scale bar represents 25 residues). Abbreviations for protein domains: GIY-YIG, amino acids that form the catalytic motif; RING, really interesting new gene. Abbreviations for organism names and UniProt identifier (in parenthesis): *H. sapiens*, *Homo sapiens* (Q9BQ83); *M. musculus*, *Mus musculus* (Q8BX32); *D. melanogaster*, *Drosophila melanogaster* (Q9VN41); *S. cerevisiae*, *Saccharomyces cerevisiae* (P38324); *S. pombe*, *Schizosaccharomyces pombe* (Q9P7M3).

The realization that Slx1 belongs to a distinct cluster of the GIY-YIG nuclease superfamily stemmed from bioinformatics analyses that identified a discrete family of URI (UvrC-intron-type) endonucleases (Aravind and Koonin, 2001), which paved the way for the identification of new members of the GIY-YIG superfamily and their evolutionary classification (Dunin-Horkawicz et al., 2006). One remarkable feature of the GIY-YIG superfamily is that the nuclease domain is almost invariably associated with other domains, fused either N- or C-terminally. The different domain architectures have allowed for the classification of GIY-YIG proteins into different lineages and subfamilies, one of which includes the Slx1 lineage (Figure 2) (Dunin-Horkawicz et al., 2006).

### 2.2 Slx4/SLX4 Is a Multi-Functional Scaffold Protein

**FIGURE 3:**
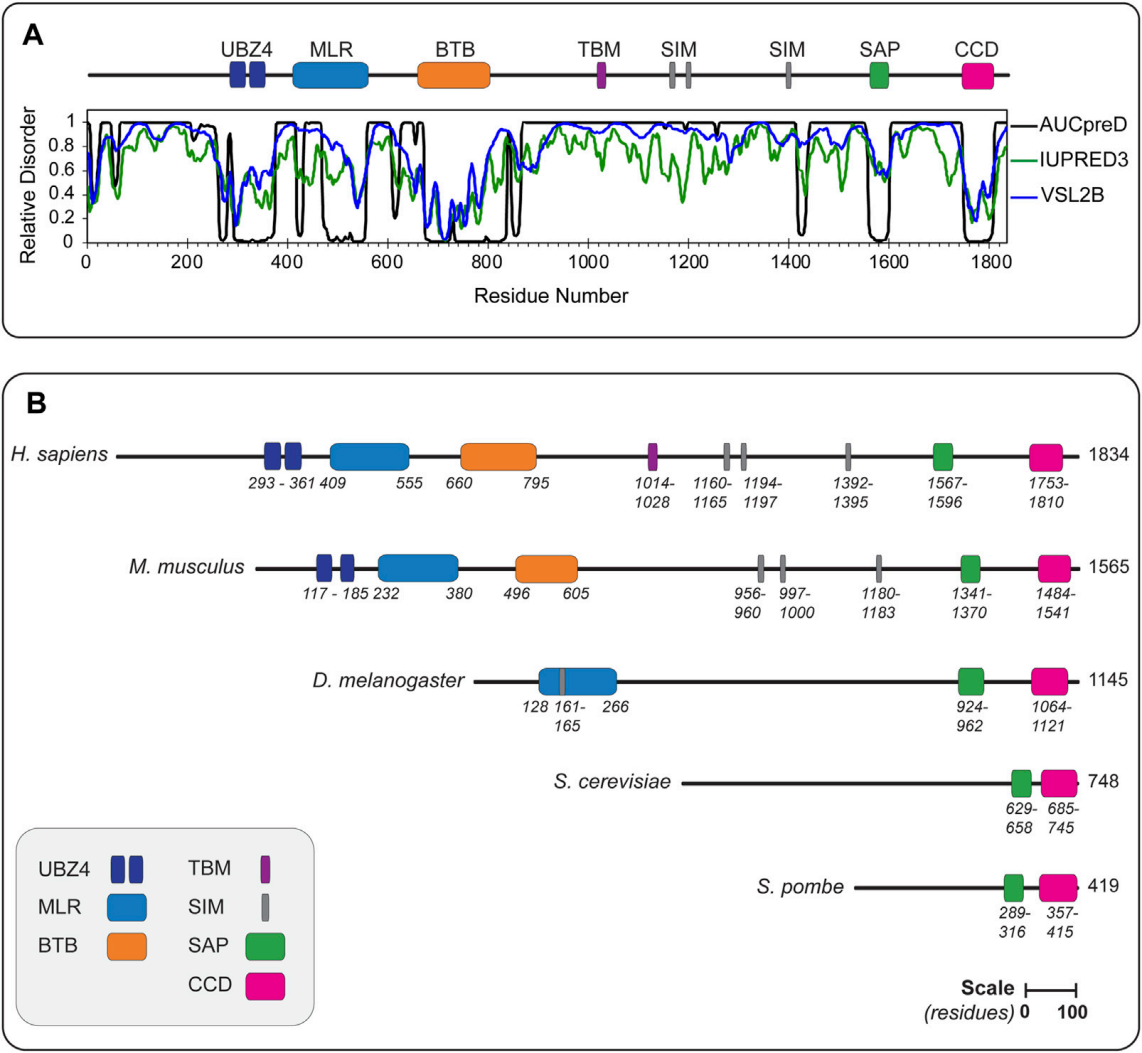
**(A)** Disorder probability plots for human SLX4 using the AUCpreD (black trace) (Wang et al., 2016), IUPRED3 (green trace) (Erdos et al., 2021) and VSL2B (blue trace) (Peng et al., 2006) predictors. These plots reveal that SLX4 is predicted to be largely disordered, as indicated by a disorder tendency score >0.5. The domain organization of human SLX4 is shown above the disorder probability plots. **(B)** The domain organization of SLX4 proteins from different eukaryotes. Domains are colored as indicated in the legend (lower left). The length of each protein is indicated in amino acids, as are the domain boundaries. All the proteins are shown on the same scale (scale bar represents 100 residues). Abbreviations for protein domains: UBZ4, ubiquitin-binding zinc finger type 4; MLR, MUS312-MEI9 interaction-like region; BTB, Broad-Complex, Tramtrack and Bric a Brac; TBM, TRF2-binding motif; SIM, SUMO-interacting motif; SAP, SAF-A/B, Acinus and PIAS; CCD, conserved C-terminal domain. Abbreviations for organism names and UniProt identifier (in parenthesis): *H. sapiens*, *Homo sapiens* (Q8IY92); *M. musculus*, *Mus musculus* (Q6P1D7); *D. melanogaster*, *Drosophila melanogaster* (Q9VS48); *S. cerevisiae*, *Saccharomyces cerevisiae* (Q12098); *S. pombe*, *Schizosaccharomyces pombe* (Q9P6M0).

In contrast to Slx1, which is conserved from yeast to human and contains well-defined structural or catalytic domains, Slx4 has diverged throughout evolution, lacks enzymatic motifs and is predicted to be predominately disordered (Figures 3A,B). In the following sections, we will highlight some of the defining features of Slx4/SLX4.

*S. pombe* Slx4 is the smallest Slx4 protein studied to date (418 amino acids). It contains the minimal domain architecture that is ubiquitous in fungi and animals: a SAF-A/B, Acinus and PIAS (SAP) domain and a conserved C-terminal domain (CCD) (Figure 3B). The SAP and CCD are connected by a flexible linker that varies in length between different organisms. As discussed in more detail below, the CCD is the most conserved region of Slx4 and consistently forms a critical part of the Slx1-Slx4 binding interface (Gaur et al., 2015; Lian et al., 2016; Gaur et al., 2019; Xu et al., 2021). By contrast, the sequence of the SAP domain varies considerably between species, which could serve different functions since the SAP domain binds DNA in *S. cerevisiae* Slx4 (Xu et al., 2021) and MUS81-EME1 in mammalian SLX4 (Fekairi et al., 2009; Svendsen et al., 2009; Castor et al., 2013; Kim et al., 2013).

One interesting difference between *S. pombe* Slx4 and *S. cerevisiae* Slx4 is that the latter contains an N-terminal extension that binds partner proteins, including two additional scaffolds called Rtt107 and Dbp11, a structure-specific DNA-binding protein called Saw1 and the Rad1-Rad10 structure-selective endonuclease. The Slx4-Rtt107-Dbp11 complex has critical roles in the DNA damage checkpoint to replication stress (reviewed in Guervilly and Gaillard, 2018). Through the interactions with Saw1 and Rad1-Rad10, Slx4 stimulates Rad1-Rad10 to remove ssDNA tails that form during a specialized form of homologous recombination called single-strand annealing (Flott et al., 2007; Li et al., 2008; Toh et al., 2010; Li et al., 2013).

The N-terminal extension of *S. cerevisiae* Slx4 is also present in animals and although the sequence varies significantly, the scaffold function is conserved. Human SLX4 has been the focus of intense research since its discovery in 2009 (Andersen et al., 2009; Fekairi et al., 2009; Munoz et al., 2009; Svendsen et al., 2009). As shown in Figure 3B, the N-terminal extension contains several core elements that mediate protein-protein interactions: two tandem ubiquitin-binding zinc finger type 4 (UBZ4) domains, the MUS312-MEI9 interaction-like region (MLR), a Broad-Complex, Tramtrack and Bric a Brac (BTB) domain, the TRF2-binding motif (TBM) and three SUMO-interacting motifs (SIM). We will explore these domains in more detail after discussing the structure and biochemical properties of a “minimal” SLX1-SLX4 heterodimer (i.e., SLX1 bound to the SLX4 CCD).

## 3 Structural and Biochemical Properties of Slx1-Slx4

Orthodox members of the SLX1 family contain a characteristic N-terminal GIY-YIG (or URI) nuclease domain and a C-terminal RING finger domain (Figure 2). As discussed below, our understanding of the structure and function of these proteins has significantly benefited from a collection of high-resolution structures of Slx1 from four different fungi, namely *Candida glabrata* (Gaur et al., 2015), *Thielavia terrestris* (Gaur et al., 2019), *S. pombe* (Lian et al., 2016) and *S. cerevisiae* (Xu et al., 2021). These structures represent snapshots of Slx1 alone (apo), Slx1 bound to Slx4 and Slx1-Slx4 co-crystallized with DNA substrates.

### 3.1 Slx1 Contains an N-Terminal GIY-YIG Nuclease Domain

**FIGURE 4:**
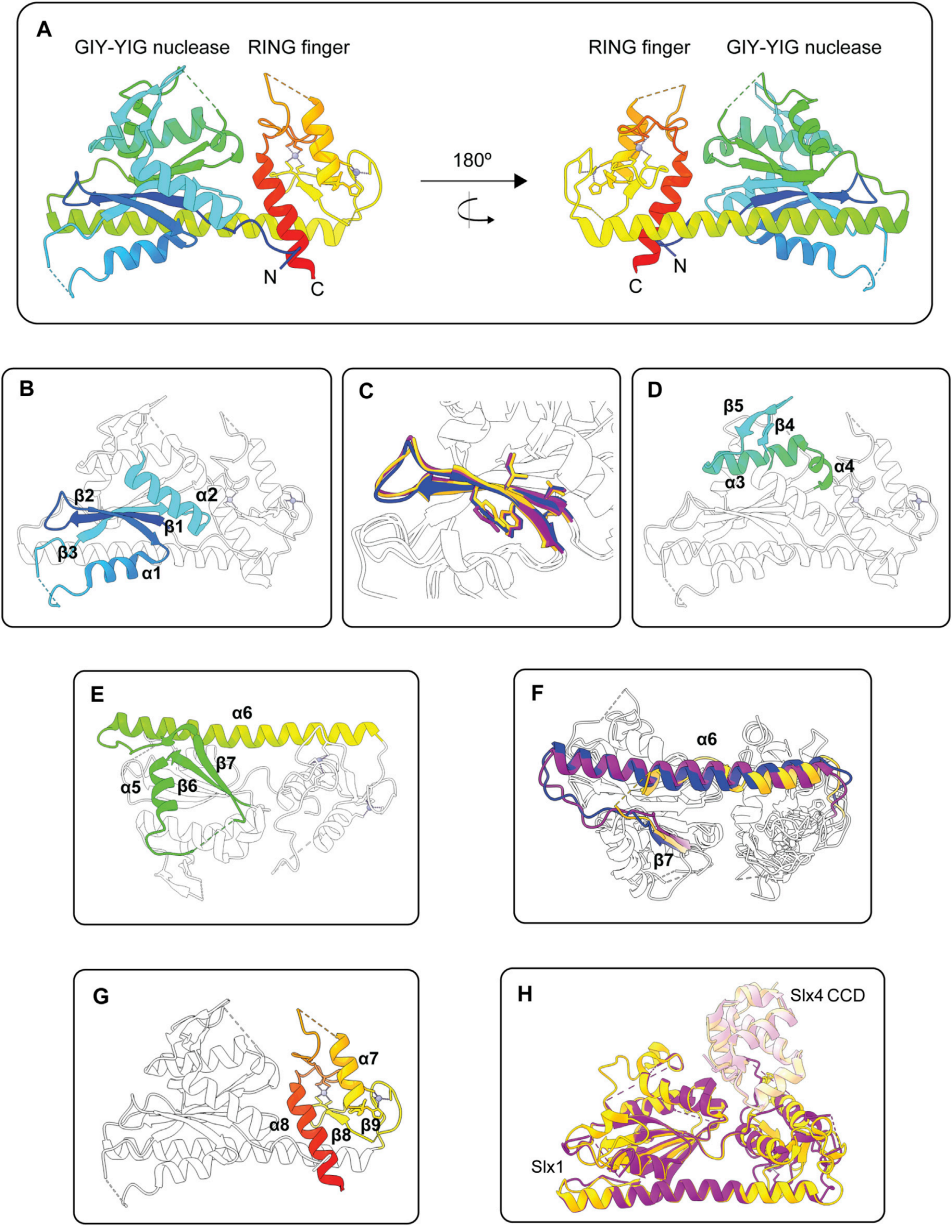
Structures of the Slx1 monomer and Slx1-Slx4 heterodimer. **(A)** Overall structure of *Candida glabrata* Slx1 shown in rainbow color (PDB: 4XM5). Zinc ions are shown as grey spheres and coordination residues are shown in stick format. Dotted lines indicate loop regions that were not visible in the electron density maps. **(B)** Structure of *C. glabrata* Slx1 showing the structural elements of the GIY-YIG nuclease domain in rainbow color (β1-β2-α1-β3-α2) and the rest of the protein in white (PDB: 4XM5). **(C)** Close-up of the GIY-YIG hairpin from *C. glabrata* Slx1 (purple; PDB: 4XLG), *Thielavia terrestris* Slx1 (blue; PDB: 6SEH) and *Saccharomyces cerevisiae* Slx1 (gold; PDB: 7CQ3). Residues forming the GIY-YIG motifs are shown in stick format. Note the subtle sequence variation in these motifs between species: GCY-YIG in *C. glabrata* Slx1, CCY-YVG in *T. terrestris* Slx1 and TVY-YIG in *S. cerevisiae* Slx1. **(D,E)** Structure of *C. glabrata* Slx1 showing features in the nuclease domain that may be unique to some Slx1 proteins in rainbow color and the rest of the protein in white (PDB: 4XM5). **(D)** A short β-hairpin (β4-β5) connects the glutamate helix (α2) to a helical segment. **(E)** The helical segment bends into strand β6, which is connected to strand β7 by α5. A linker connects β7 to a long α-helix (α6) that essentially provides a scaffold for the GIY-YIG nuclease and RING finger domains. Note that the protein shown in **(E)** has been rotated vertically by approximately 180° compared to **(D)**. **(F)** Overlay showing that the length of helix α6 varies between Slx1 from *C. glabrata* (purple; PDB: 4XLG), *T. terrestris* (blue; PDB: 6SEH) and *S. cerevisiae* Slx1 (gold; PDB: 7CQ3). Strand β7 is also shown in color. **(G)** Structure of *C. glabrata* Slx1 showing the structural elements of the RING domain in rainbow color (β8-β9-α7-α8) and the rest of the protein in white (PDB: 4XM5). **(H)** Overlay of crystal structures of Slx1 bound to fragments of Slx4 that contain the CCD. *C. glabrata* Slx1-Slx4^CCD^ is shown in purple (PDB: 4XLG) and *S. cerevisiae* Slx1-Slx4^CCD^ is shown in gold (PDB: 7CQ3), where darker shades represent Slx1 and lighter shades represent Slx4^CCD^. Abbreviations: GIY-YIG, amino acids that form the catalytic motif; RING, really interesting new gene; CCD, conserved C-terminal domain.

The published structures of Slx1 collectively reveal that the GIY-YIG nuclease domain contains a five-stranded anti-parallel β-sheet flanked by several α-helices (Gaur et al., 2015; Gaur et al., 2019; Xu et al., 2021) (Figure 4A). Several of the expected core elements are present, including the GIY-YIG hairpin formed by strands β1 and β2, an arginine helix (α1) that contains an invariant Arg residue, a linker strand that extends the GIY-YIG hairpin into an anti-parallel β-sheet and the glutamate helix (α2), which contains a conserved Glu residue (Figure 4B). The Slx1 active site residues are in the GIY-YIG hairpin, with the first motif (GIY) found in β1 and the second motif (YIG) found in β2 (Figure 4C). We will revisit the GIY-YIG motif when we discuss the catalytic mechanism of Slx1.

The apo structure of *C. glabrata* Slx1 revealed additional features in the nuclease domain that may be unique to some Slx1 proteins. First, a short β-hairpin (β4-β5) connects the glutamate helix (α2) to a helical segment (Figure 4D) (Gaur et al., 2015). The helical segment bends into strand β6, which is connected to strand β7 by an α-helix (Figure 4E). Notably, the β-hairpin was not observed in the Slx1-Slx4 structures, suggesting that this region adopts a different conformation in the presence of Slx4 (Gaur et al., 2015; Gaur et al., 2019; Xu et al., 2021). The second notable feature is a long α-helix (α6 in *C. glabrata* Slx1) that essentially provides a scaffold for the GIY-YIG nuclease and RING finger domains (Gaur et al., 2015) (Figures 4A,E). Intriguingly, the length of this helix varies between Slx1 from different fungi (Figure 4F). It would be interesting to determine whether this α-helix regulates the folding, stability, or enzymology of Slx1-Slx4. For example, one could substitute the short α-helix of *T. terrestris* Slx1 with the long α-helix from *C. glabrata* or *S. cerevisiae* Slx1 and then compare the biophysical and enzymatic properties of the mutant enzymes to that of wild type Slx1-Slx4. Another curious observation is that the helix is replaced with an extended unstructured region in human SLX1 (Jumper et al., 2021). The functional implications of this divergence are unknown, and the field anxiously awaits high-resolution structures of human SLX1-SLX4.

### 3.2 The C-Terminal RING Finger Domain

The C-terminal RING finger domain contains two α-helices and one or two anti-parallel β-sheets (Figures 4A,G) (Gaur et al., 2015; Lian et al., 2016; Gaur et al., 2019; Xu et al., 2021). There are also two zinc ions bound in the RING finger domain of all published Slx1 structures (Gaur et al., 2015; Lian et al., 2016; Gaur et al., 2019; Xu et al., 2021). Sequence and structural analyses show that the Slx1 RING finger domain is an atypical C4HC3-type RING finger (Gaur et al., 2015; Lian et al., 2016). Although many proteins containing this domain function as E3 ubiquitin ligases, the Slx1 RING finger domain appears to be required for intermolecular protein contacts that stabilize the Slx1-Slx4 heterodimer or, in the absence of Slx4, an Slx1 homodimer (Gaur et al., 2015; Lian et al., 2016; Gaur et al., 2019; Xu et al., 2021). Of note, the C-terminal tail of *S. pombe* Slx1 contains a SIM that binds Pmt3 (*S. pombe* SUMO) *in vitro*, raising the possibility that SUMOylated proteins could help recruit Slx1-Slx4 to specific DNA lesions *in vivo* (Lian et al., 2016).

### 3.3 Insights Into the Mechanism of Slx1-Slx4 Heterodimerization

Our mechanistic understanding of Slx1-Slx4 heterodimerization has significantly benefitted from crystal structures of full-length Slx1 bound to C-terminal fragments of Slx4 that contain the Slx1-binding domain (Gaur et al., 2015; Gaur et al., 2019; Xu et al., 2021). This region of Slx4 is conserved among animals and is therefore referred to as the conserved CCD. The overall structures of *C. glabrata*, *T. terrestris* and *S. cerevisiae* Slx1-Slx4^CCD^ are very similar (Figure 4H) (Gaur et al., 2015; Gaur et al., 2019; Xu et al., 2021). The most variable regions are in Slx1 and include: i) the loop between the arginine helix (α1) and β3 (the linker strand), ii) the loop between the glutamate helix (α2) and α3, and iii) the RING finger domain. Additionally, the β4-β5 hairpin was not observed in the Slx1-Slx4^CCD^ structures. The Slx4 CCD contains five short α-helices that fold into a globular domain (Gaur et al., 2015; Gaur et al., 2019; Xu et al., 2021). The SLX4^CCD^ fits into a cleft located between the Slx1 nuclease and RING domains (Figure 4H). Two main regions define the Slx1-Slx4^CCD^ binding interface. Region 1 involves hydrophobic contacts between Slx4^CCD^ α2 and the Slx1 RING finger domain; these interactions bury 556 Å of surface area in *S. cerevisiae* Slx1-Slx4^CCD^. In contrast, Region 2 involves polar interactions between Slx4^CCD^ α5 and the Slx1 nuclease domain, which bury 520 Å of surface area.

As mentioned above, Slx1-Slx4 heterodimerization is a prerequisite for catalytic activity *in vitro*. In the absence of Slx4, *C. glabrata* Slx1 forms a salt-resistant homodimer that partially blocks the active site and DNA-binding residues (Gaur et al., 2015). Analysis of the crystal structures reveals that the position of one Slx1 monomer overlaps with Slx4^CCD^ in the Slx1-Slx4^CCD^ structure, indicating mutually exclusive protein-protein interactions. Indeed, the transition from homodimer to heterodimer is promoted by Slx4^CCD^ (Gaur et al., 2015). Two important and unresolved questions are whether monomeric Slx1 exhibits nuclease activity and whether *C. glabrata* Slx1 self-associates *in vivo*. It will be equally insightful to know whether this biochemical property is conserved through evolution. The ability of Slx1 to self-associate could also reflect the first principle of protein folding: globular proteins fold by minimizing the nonpolar surface exposed to water. Although we do not know whether Slx1 self-associates *in vivo*, we speculate that monomeric Slx1 is not stable in the absence of Slx4 and is likely targeted for proteasomal degradation. This may explain why the loss of SLX4 in human and murine cells leads to a significant or complete reduction in SLX1 levels (Munoz et al., 2009; Castor et al., 2013; Kim et al., 2013; Wyatt et al., 2013; Sarbajna et al., 2014). It would be interesting to determine whether the residual SLX1 in these cells exists as a self-inhibited homodimer and, if so, the biological consequences of expressing a constitutively monomeric nuclease.

### 3.4 Mechanisms of DNA-Binding and Cleavage

Slx1-Slx4 is unique among structure-selective endonucleases because it cleaves all types of branched DNA structures, including replication and early recombination intermediates (e.g., replication forks, splayed arms, 5′- and 3′-flaps), as well as late recombination intermediates (e.g., nicked and intact Holliday junctions) (Figure 1A). Structural, biochemical and computational analyses of *C. glabrata* and *T. terrestris* Slx1-Slx4^CCD^ revealed that Slx1 has three different positively charged patches that bind branched DNA substrates, designated as sites I–III (Gaur et al., 2015; Gaur et al., 2019). Site I contains conserved residues from the arginine helix that engage dsDNA on one side of the branchpoint (i.e., the pre-nick DNA duplex). When reconstituted with Slx4^CCD^, Slx1 site I mutants exhibited modest defects in substrate binding but showed severe catalytic defects (Gaur et al., 2015; Gaur et al., 2019). This suggests that site I is a low-affinity binding site that has an important role in orientating the substrate for catalysis. Site II contains residues from the glutamate helix that interact with ssDNA. When co-purified with Slx4^CCD^, Slx1 site II mutants showed moderate to severe defects in substrate binding and catalysis (Gaur et al., 2015; Gaur et al., 2019). Finally, site III contains residues from three elements: the GIY-YIG hairpin loop, a short helix that connects the arginine helix to β3 and a flexible loop that connects the glutamate helix to α3. A comparison of the apo and DNA-bound structures of *T. terrestris* Slx1-Slx4^CCD^ showed that the loop becomes ordered in the presence of DNA (Gaur et al., 2019). The proximity of this region to the active site suggests that the ordering of site III is coupled to active site assembly. As such, site III seems to represent a molecular safety latch, ensuring that catalysis only occurs if the DNA substrate is bound correctly in sites I and III (Gaur et al., 2019). Importantly, Slx1-Slx4^CCD^ variants with mutations in sites II and III showed a weak affinity for DNA substrates and were catalytically inactive (Gaur et al., 2019). This indicates that substrate binding in site I alone is insufficient for enzyme activity. A further implication is that sites II and III are required to hold the DNA substrate in place and facilitate or guide incision site selection. Intriguingly, the orientation of site III relative to site I is such that the DNA substrate bends by approximately 50° for efficient catalysis (Gaur et al., 2019). Notably, the FEN1 and Mus81-Mms4/MUS81-EME1 structure-selective endonucleases also use DNA bending to achieve optimal positioning of the substrate in the active site (Tsutakawa et al., 2011; Gwon et al., 2014; Mukherjee et al., 2014).

In a recent development, Xu et al. published the crystal structures of *S. cerevisiae* Slx1 in complex with an Slx4 fragment containing the SAP and CCD domains (Slx1-Slx4^SAP+CCD^), alone and bound to a 1-nt 5′-flap substrate (Xu et al., 2021). The overall structure of Slx1-Slx4^SAP+CCD^ was very similar in the apo and DNA-bound forms with one notable exception: the SAP domain was only visible in the presence of DNA, indicating that it adopted a more ordered conformation in the DNA-bound state. More specifically, the SAP domain folded into three helices, in which α2 and α3 packed against the Slx4 CCD domain (Xu et al., 2021). Although this structure captured Slx1-Slx4^SAP+CCD^ in a catalytically-inhibited conformation, it provided the framework for molecular dynamics simulations of Slx1-Slx4^SAP+CCD^ bound to a longer 5′-flap, which agree well with the model predicted for the *T. terrestris* Slx1-Slx4^CCD^-DNA complex (Gaur et al., 2019; Xu et al., 2021). Together, these models reveal three critical regions of protein-DNA contacts: i) Slx1 site I binds the major groove of dsDNA on one side of the branchpoint (i.e., the pre-nick DNA duplex) and this is essential to place the scissile phosphate in the active site; ii) the Slx4 SAP domain binds the minor groove of the other DNA duplex (i.e., post-nick DNA duplex) approximately one turn away from the ss/ds-DNA branchpoint; and iii) Slx1 sites II and III engage the ssDNA flap, with site III becoming more critical for binding longer flaps. Curiously, the SAP domain mediates the interaction between mammalian SLX4 and MUS81 (Fekairi et al., 2009; Svendsen et al., 2009; Castor et al., 2013; Kim et al., 2013), suggesting that its function may have diverged from DNA-binding to protein-binding in some species. Another possibility is that the SAP domain uses distinct interfaces to accommodate DNA and protein substrates simultaneously.

One limitation to the models described above is that they do not account for the incision site selection of Slx1-Slx4; the phosphodiester bond that is closest to the active site does not match what is cleaved by Slx1-Slx4 *in vitro* (i.e., between nucleotides 3 and 4 on the 3′-side of the ss/ds-DNA junction) (Gaur et al., 2019; Xu et al., 2021). Further structural studies are needed to capture the enzyme in a catalytically productive conformation. Nevertheless, a catalytic mechanism has been proposed for Slx1. As mentioned above, the Slx1 active site residues are in the GIY-YIG hairpin, with the first motif (GIY) found in β1 and the second motif (YIG) found in β2 (Figure 4C). Although both motifs exhibit subtle sequence variation, the Tyr residues are strongly conserved between GIY-YIG nucleases. This led Gaur et al. (2015) to propose that the catalytic mechanism of Slx1 is the same as that described by Sokolowska et al. (2011) for the Hpy188I restrictase. Briefly, phosphodiester hydrolysis involves an in-line nucleophilic attack of the scissile phosphate. Catalysis requires a divalent metal ion in the active site. The tyrosine residue in the GIY motif (Y14 in *C. glabrata* Slx1) serves as a general base, acting as a proton acceptor for the nucleophilic water molecule (Sokolowska et al., 2011; Gaur et al., 2015). In *C. glabrata* Slx1, the guanidinium group of invariant R36 and the phenolic oxygen of Y26 stabilize the non-bridging oxygen of the scissile phosphate. A conserved glutamate residue (E79 in *C. glabrata* Slx1) anchors a single metal cation in the active site (Sokolowska et al., 2011; Gaur et al., 2015). The metal ion is located on the opposite side of the scissile phosphate and is thought to destabilize the substrate and stabilize the transition state.

## 4 SLX1-SLX4 Has Pivotal Roles in DNA Repair and Genome Stability

Slx1-Slx4/SLX1-SLX4 is a unique structure-selective endonuclease because of its ability to cleave a wide range of branched DNA structures. This confers the enzyme with pivotal roles in the maintenance of genome stability, including the cellular response to replication stress, telomere homeostasis and multiple DNA repair pathways (i.e., homologous recombination and interstrand crosslink repair). The role of Slx1-Slx4/SLX1-SLX4 in homologous recombination is conserved from yeast to human (Mullen et al., 2001; Fricke and Brill, 2003; Coulon et al., 2004; Zhang et al., 2006; Andersen et al., 2009; Fekairi et al., 2009; Munoz et al., 2009; Svendsen et al., 2009; Andersen et al., 2011; Saito et al., 2012; Agostinho et al., 2013; Castor et al., 2013; Garner et al., 2013; Wyatt et al., 2013; Sarbajna et al., 2014). Human SLX1-SLX4 fulfills additional roles because the SLX4 scaffold interacts with a plethora of proteins, including two additional structure-selective endonucleases (i.e., XPF-ERCC1 and MUS81-EME1), telomere-binding proteins (e.g., TRF2-RAP1), cell cycle control factors (e.g., PLK1, TOPBP1), as well as ubiquitin and SUMO (Andersen et al., 2009; Fekairi et al., 2009; Munoz et al., 2009; Svendsen et al., 2009; Guervilly and Gaillard, 2018). As discussed below, the N-terminal extension of human SLX4 contains several domains or motifs that regulate some of these protein-protein interactions.

### 4.1 Human SLX4 Contains an MLR Domain That Binds XPF-ERCC1 and SLX4IP

**FIGURE 5:**
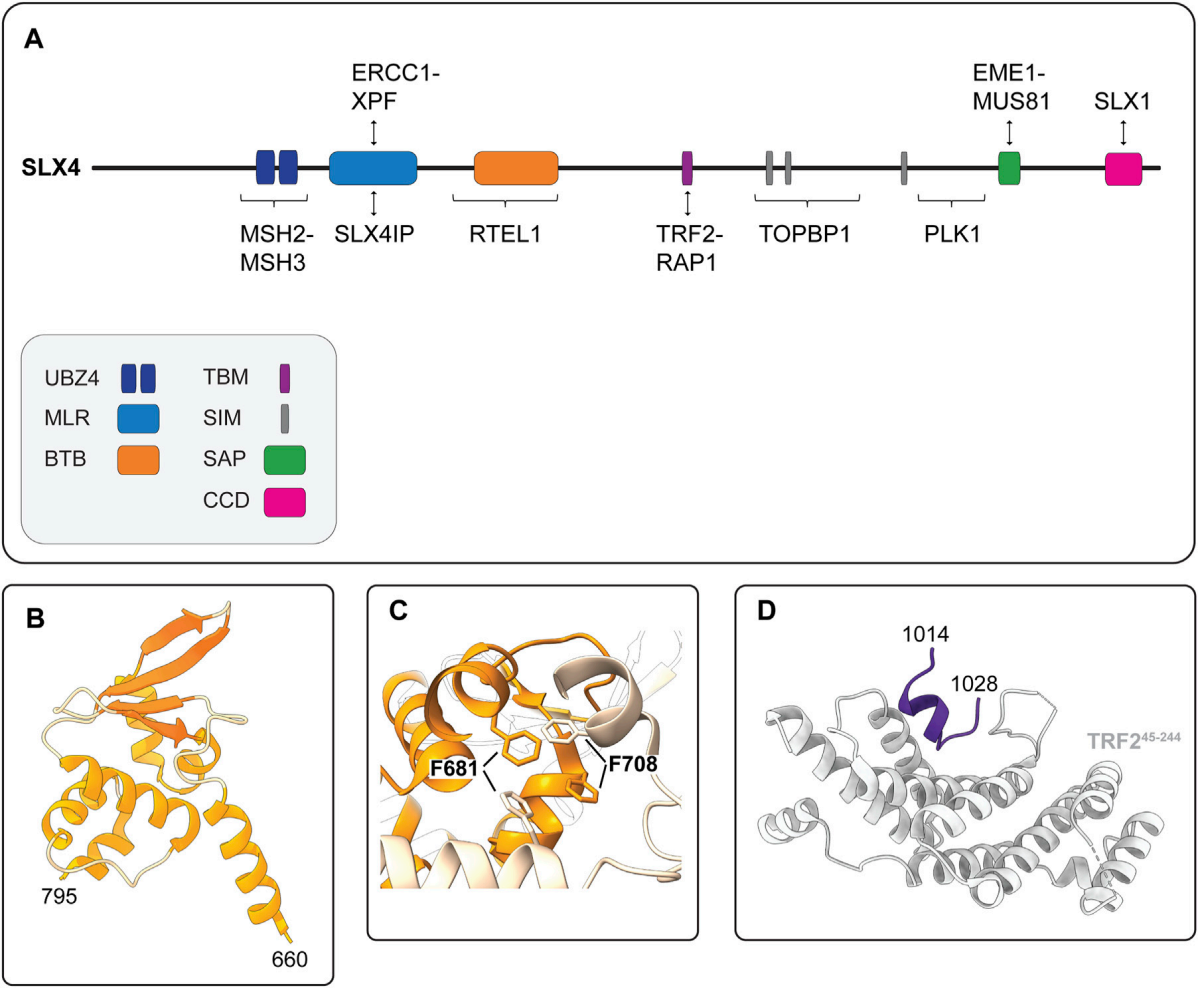
Human SLX4 is a scaffold for proteins that have critical functions in DNA repair and genome stability. **(A)** Schematic representation of human SLX4 showing its domain organization and select protein partners. Domains are colored as indicated in the legend (lower left). Abbreviations for protein domains: UBZ4, ubiquitin-binding zinc finger type 4; MLR, MUS312-MEI9 interaction-like region; BTB, Broad-Complex, Tramtrack and Bric a Brac; TBM, TRF2-binding motif; SIM, SUMO-interacting motif; SAP, SAF-A/B, Acinus and PIAS; CCD, conserved C-terminal domain. **(B)** Crystal structure of the human SLX4 BTB domain, spanning residues 660–795 (PDB: 4ZOU). **(C)** Close-up view of the critical hydrophobic interactions between F681 and F708 in the SLX4 BTB dimerization interface (PDB: 4ZOU). One monomer is shown in orange and the other is shown in peach. Residues F681 and F708 are shown in stick format. **(D)** Crystal structure of the human SLX4 TBM (shown in purple) bound to a fragment of TRF2 spanning residues 45–244 (shown in grey) (PDB: 4M7C).

The N-terminal extension of human SLX4 contains several domains or motifs that bind functionally diverse proteins, most of which have key roles in maintaining genome stability (Figure 5A). The MUS312-MEI9 interaction-like region, spanning residues 500–559, mediates the interaction between SLX4 and XPF-ERCC1 (Fekairi et al., 2009; Svendsen et al., 2009; Kim et al., 2013; Guervilly et al., 2015; Hashimoto et al., 2015). Residues L530, F545, Y546 and L550 are crucial for the SLX4-XPF interaction in yeast two-hybrid (Hashimoto et al., 2015) and coimmunoprecipitation assays (Guervilly et al., 2015; Zhang et al., 2019), suggesting that these residues form part of the binding interface. More recently, the MLR domain was shown to bind a protein called SLX4IP (for SLX4-interacting protein). We will revisit the structural anatomy and functions of these different SLX4-complexes in the final part of this review (Section 6.1).

### 4.2 Some Assembly Required: Human SLX4 Contains a BTB Domain That Confers Self-Association

Another important element of human SLX4 is the BTB domain, which is positioned downstream of the MLR domain and spans residues 660–795 (Figure 5A). The BTB domain is a widely distributed motif that participates in diverse cellular functions, ranging from transcriptional regulation to ion channel assembly and gating (Stogios et al., 2005). In general, BTB domains mediate self-oligomerization (e.g., homodimerization) or interactions with other proteins. Structural, biochemical and cell-based experiments indicate that the SLX4 BTB domain promotes homodimerization (Guervilly et al., 2015; Yin et al., 2016). The crystal structure of the BTB dimer revealed that each monomer adopted the characteristic ΒΤΒ fold—a cluster of six α-helices capped at one end by a four-stranded β-sheet (Figure 5B) (Yin et al., 2016). The dimer was held together by a hydrophobic interface with key contacts made between residues in α1 and α2, most notably residues F681 and F708 (Figure 5C). These residues are conserved in animals, suggesting that BTB-mediated SLX4 homodimerization may be a common theme amongst metazoans (Yin et al., 2016).

Notably, the BTB domain is needed for interstrand crosslink repair, which may reflect its role in promoting XPF SUMOylation and stabilizing the interaction between XPF and SLX4 (Andersen et al., 2009; Kim et al., 2013; Guervilly et al., 2015). As discussed in more detail below, the BTB domain also mediates several SLX4 functions in cells that maintain telomere lengths by the ALT (alternative lengthening of telomeres) pathway. These functions include the formation of subnuclear foci, the recruitment of SLX1 and XPF-ERCC1 to telomeres and telomere stability (Yin et al., 2016). Further studies are needed to elucidate how the BTB domain regulates the functions of different SLX4-nuclease complexes.

### 4.3 The Telomere Connection: Human SLX4 Contains a TRF2-Binding Motif

As alluded to above, SLX4 has an important role in maintaining telomere length and stability. This is not surprising, given that branched DNA structures are expected to occur frequently in telomeres because of their highly repetitive DNA sequence (for reviews, see Maestroni et al., 2017; Bonnell et al., 2021). Telomeres are essential nucleoprotein structures that form a protective cap for the terminal segments of linear chromosomes. In humans, the DNA component contains hundreds of hexameric repeats (5′-TTAGGG-3′) organized into a centromere-proximal double-stranded region and a single-stranded 3′-overhang. Several histone and non-histone proteins bind telomeric DNA. In mammalian cells, a group of six non-histone proteins (TRF1, TRF2, RAP1, POT1, TPP1 and TIN2) form a macromolecular protein complex called shelterin, which has a crucial role in telomere protection (reviewed in Palm and De Lange, 2008; Lim and Cech, 2021). TRF2 aids in the formation of DNA secondary structures called telomere loops (t-loops), in which the single-stranded overhang loops back on itself and the duplex region to form a lariat-like structure (Griffith et al., 1999; Doksani et al., 2013). The t-loop provides an architectural mechanism to “hide” the single-stranded overhang and prevent chromosome ends from being recognized as broken DNA (reviewed in Lim and Cech, 2021). Although the t-loop protects the chromosome end, this structure must be protected from other enzymes in the cell. For example, the base of the t-loop represents a branched DNA substrate that can be cleaved by SLX1-SLX4, leading to telomere loss. TRF2 has emerged as the gatekeeper that regulates the accessibility of telomeres to structure-selective endonucleases to prevent the unscheduled cleavage of branched DNA structures (Poulet et al., 2009; Saint-Leger et al., 2014; Sarkar et al., 2015; Schmutz et al., 2017).

One of the hallmarks of cancer cells is their replicative immortality, achieved by activating a telomere maintenance mechanism (Hanahan and Weinberg, 2011). Most cancer cells reactivate a reverse transcriptase called telomerase, but a minority use the ALT mechanism (Kim et al., 1994; Bryan et al., 1995; Bryan et al., 1997; Shay and Bacchetti, 1997; MacKenzie et al., 2021). ALT is a homology-directed recombination-based pathway that uses telomeric templates for DNA synthesis (Dunham et al., 2000; MacKenzie et al., 2021). The recruitment of SLX4 to ALT telomeres requires the BTB domain (discussed above), as well as the interaction with TRF2 (Fekairi et al., 2009; Munoz et al., 2009; Svendsen et al., 2009; Wan et al., 2013; Wilson et al., 2013; Yin et al., 2016). SLX4 uses a unique TBM to interact directly with TRF2 (Wan et al., 2013; Wilson et al., 2013). This motif includes residues 1,014–1,028, which are positioned downstream of the BTB domain in a largely disordered region of SLX4 (Figure 3A, 5A). Conversely, SLX4 binds to the TRF homology (TRFH) domain of TRF2 (Wan et al., 2013). The TRFH domain is a versatile docking site for various partner proteins (Giraud-Panis et al., 2018). The crystal structure of the SLX4^TBM^-TRF2^TRFH^ complex shows that SLX4^TBM^ adopts an extended conformation with a short one-turn helix at the N-terminus and fits neatly into a narrow groove formed by the TRF2^TRFH^ (Figure 5D) (Wan et al., 2013). The interface is hydrophobic and includes three residues from SLX4^TBM^ (H1020, L1022, P1024) and one from TRF2^TRFH^ (F120). Through the interaction with TRF2, SLX4 and its associated nucleases, most notably SLX1, prevent telomeric DNA damage and fragility in ALT and non-ALT cells (Wilson et al., 2013; Saint-Leger et al., 2014; Sarkar et al., 2015; Yin et al., 2016; Yang et al., 2020). In ALT cells, SLX4 has an additional role in regulating telomere length. ALT telomeres are unusually long and heterogeneous and contain a greater abundance of TRF2 and SLX4 than non-ALT cells (Dejardin and Kingston, 2009; Svendsen et al., 2009; Wan et al., 2013; Wilson et al., 2013). Here, SLX4 promotes telomere recombination and negatively regulates telomere length, most likely *via* SLX1-mediated cleavage of recombination intermediates (Wan et al., 2013; Sarkar et al., 2015; Sobinoff et al., 2017). The actions of SLX4 and its associated endonucleases are essential for “productive ALT,” which refers to an equilibrium between pro- and anti-recombinogenic activities, such that telomere lengths are maintained at or near a threshold compatible with cell growth (Sarkar et al., 2015; Sobinoff et al., 2017; Panier et al., 2019; Robinson et al., 2020). We will revisit the ALT mechanism later in this review.

### 4.4 It Takes Two to Tango: Human SLX4 Binds Ubiquitin and SUMO

Human SLX4 has acquired the capacity to recognize ubiquitin through two tandem UBZ4 domains in the N-terminus (Figure 5A), referred to as UBZ-1 and UBZ-2, respectively (Fekairi et al., 2009; Kim et al., 2011; Lachaud et al., 2014). Ubiquitin is a small, ubiquitously expressed regulatory protein initially characterized as an essential component of the ATP-dependent proteolytic system (reviewed in Swatek and Komander, 2016). Today, the covalent modification of proteins with ubiquitin is understood to represent a versatile signaling event that impacts hundreds of cellular processes, including the recruitment and activity of DNA repair complexes (Tang et al., 2021).

The first clue that the SLX4 UBZ4 domains were physiologically relevant came from identifying Fanconi anemia patients carrying in-frame deletions of *SLX4* that disrupted UBZ-1 and deleted UBZ-2 (Kim et al., 2011; Stoepker et al., 2011). Further studies showed that the UBZ domains were uniquely required for the survival of cells treated with DNA interstrand crosslinking agents (Kim et al., 2013; Ouyang et al., 2015). Around the same time, another group showed that UBZ domains of chicken DT40 SLX4 were required for cellular tolerance to interstrand crosslinking agents (Yamamoto et al., 2011). This study also revealed that the SLX4 was recruited to DNA damage-induced foci through an interaction between the UBZ domains and monoubiquitylated FANCD2. Notably, FANCD2 monoubiquitination traps the FANCI-FANCD2 complex on DNA, which likely regulates the recruitment of downstream proteins (Alcon et al., 2020; Rennie et al., 2020; Tan et al., 2020; Wang et al., 2020; Wang et al., 2021). However, it is still not clear whether the UBZ domains of human SLX4 bind monoubiquitinated FANCD2. First, *in vitro* ubiquitin-binding assays showed that the isolated UBZ domains bound K63-linked polyubiquitin chains but not monoubiquitin (Kim et al., 2011). Further work showed that UBZ-1 was necessary and sufficient for binding to polyubiquitin chains but exhibited weak binding to monoubiquitin (Lachaud et al., 2014). Second, SLX4 was efficiently recruited to interstrand crosslinks in cells that lacked FANCD2 or its E3 ubiquitin ligase RAD18 (Lachaud et al., 2014). Together, these data suggest that monoubiquitylated FANCD2 is not the primary ligand for SLX4 UBZ-1. Nevertheless, there are reports that SLX4 and FANCD2 colocalize in subnuclear foci under certain cellular conditions (Minocherhomji et al., 2015; Panichnantakul et al., 2021). Further studies are needed to determine the ubiquitylated ligand(s) that recruit SLX4 to DNA interstrand crosslinks.

Another interesting property of human SLX4 is its ability to interact non-covalently with SUMO *via* three SIMs (Figure 5A) (Guervilly et al., 2015; Ouyang et al., 2015). Like ubiquitin, SUMO is a key regulator of the DNA damage response and coordinates the recruitment of many DNA repair proteins (Morris and Garvin, 2017). Immunoprecipitation experiments showed that SLX4 captured SUMO-1 and SUMO-2/3 conjugates from cell extracts, but not free SUMO (Guervilly et al., 2015; Ouyang et al., 2015). Further work revealed that SLX4 preferentially bound SUMO-2/3 chains *in vitro* (Ouyang et al., 2015). In all cases, the SIM domains mediated SLX4-SUMO interactions (Guervilly et al., 2015; Ouyang et al., 2015). Molecular studies showed that the SIMs and UBZs have nonredundant roles in the suppression of chromosomal instability. While the UBZs have a critical role in DNA interstrand crosslink repair, the SIMs are required for the localization of SLX4 to telomeres and sites of DNA damage, as well as its role in the general replication stress response (Kim et al., 2013; Gonzalez-Prieto et al., 2015; Guervilly et al., 2015; Ouyang et al., 2015). Although the SUMOylated targets that SLX4 recognizes remain largely uncharacterized, two strong candidates are XPF and the shelterin protein RAP1 (Guervilly et al., 2015; Robinson et al., 2021). Future studies are needed to dissect the mechanism of XPF SUMOylation. We will revisit the mechanism and functions of SUMOylated RAP1 in the final section of this review.

Before concluding our discussion of SLX4 architecture, it is worth noting that Guervilly et al. (2015) showed that the SLX4 SIMs mediate its interaction with the SUMO E2-conjugating enzyme UBC9. Additionally, SLX4 protein levels were positively correlated with XPF SUMOylation *in vivo* (Guervilly et al., 2015). This raised the possibility that SLX4 was either a SUMO E3 ligase or an essential component of a macromolecular SUMO E3 ligase complex. Although Guervilly et al. (2015) did not detect E3 ligases in their SLX4 immunoprecipitates, recent work has captured interactions between SLX4 and several E3 ligases, including MMS21 and PIAS1 (Robinson et al., 2021, our unpublished data). This new data indicates that SLX4 is likely functioning as a scaffold to bridge the interactions between SUMO E2 and E3 enzymes and their targets.

## 5 Human SLX4 Is the Scaffold for Macromolecular Structure-Selective Endonuclease Complexes

Human SLX4 is a molecular scaffold for proteins that have critical roles in DNA repair and the maintenance of genome stability. To date, the roles of SLX4 in genome stability are best understood within the context of its associated structure-selective endonucleases: SLX1, MUS81-EME1 and XPF-ERCC1 (for a review, see Nowotny and Gaur, 2016). For example, human SLX4 stimulates the catalytic activity of SLX1 and MUS81-EME1 and relaxes the substrate specificity of MUS81-EME1 (Svendsen et al., 2009; Wyatt et al., 2017). Likewise, murine SLX4 stimulates the activity of XPF-ERCC1 (Hodskinson et al., 2014). Through these functions, SLX4 coordinates the removal of branched DNA structures that form in response to replication stress, the resolution of recombination intermediates and the repair of DNA interstrand crosslinks (for reviews, see Guervilly and Gaillard, 2018; Young and West, 2021).

As discussed above, most of the cellular SLX1 is degraded in the absence of SLX4 and therefore, we consider SLX1 to be a constitutive SLX4-binding partner. We also assume that all the cellular SLX4 is bound to SLX1, although this has not been tested rigorously and there are conflicting reports about SLX4 stability in the absence of SLX1 (Munoz et al., 2009; Wyatt et al., 2013; Panier et al., 2019). Based on these assumptions, human cells appear to contain two different macromolecular nuclease complexes: a di-nuclease complex composed of SLX1-SLX4 and XPF-ERCC1 (i.e., SX) and a tri-nuclease complex composed of SLX1-SLX4, MUS81-EME1 and XPF-ERCC1 (i.e., SMX). Current data indicate that the SMX complex is formed predominately in mitosis. The molecular assembly and functions of these complexes are discussed below.

### 5.1 The SX Di-Nuclease Complex

**FIGURE 6:**
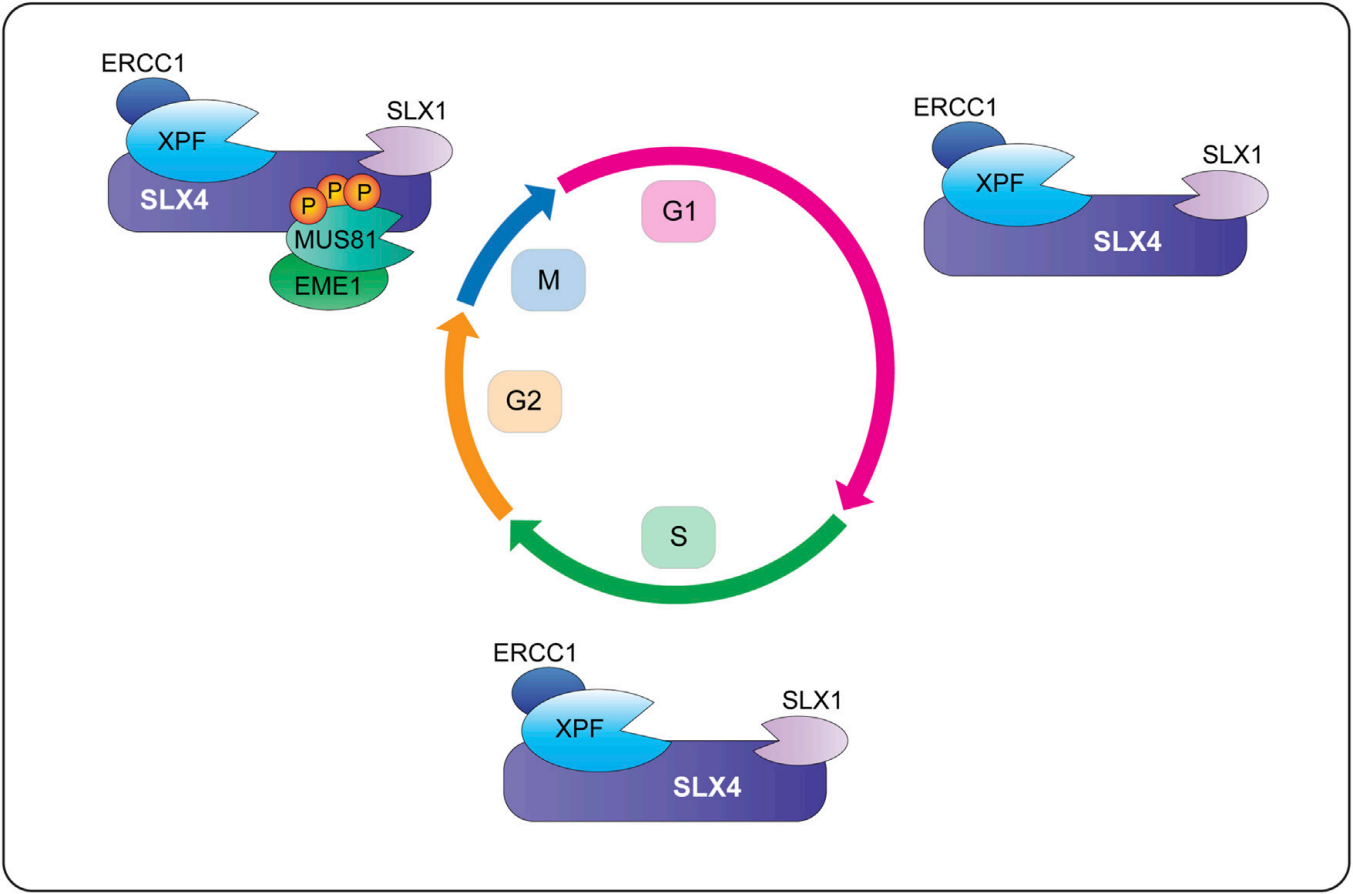
Temporal regulation of SLX4-nuclease complexes in human cells. During G1 and S-phase, SLX1-SLX4 is bound to XPF-ERCC1 and forms a di-nuclease complex called SX (for the catalytic subunits SLX1 and XPF). Cells also contain pools of XPF-ERCC1 and MUS81-EME1 that are not bound to SLX1-SLX4 (not shown). When cells enter mitosis, CDK1 phosphorylates SLX4 and CK2 phosphorylates MUS81. These phosphorylation events promote the recruitment of MUS81-EME1 onto the SLX4 scaffold, leading to the formation of a tri-nuclease complex called SMX (for the catalytic subunits SLX1, MUS81 and XPF). More work is needed to determine whether mitotic cells contain SX and SMX complexes or pools of XPF-ERCC1 and MUS81-EME1 that are not bound to SLX1-SLX4 (not shown). Abbreviations: G1, gap 1; S, DNA synthesis; G2, gap 2; M, mitosis; P, phosphorylation.

**FIGURE 7:**
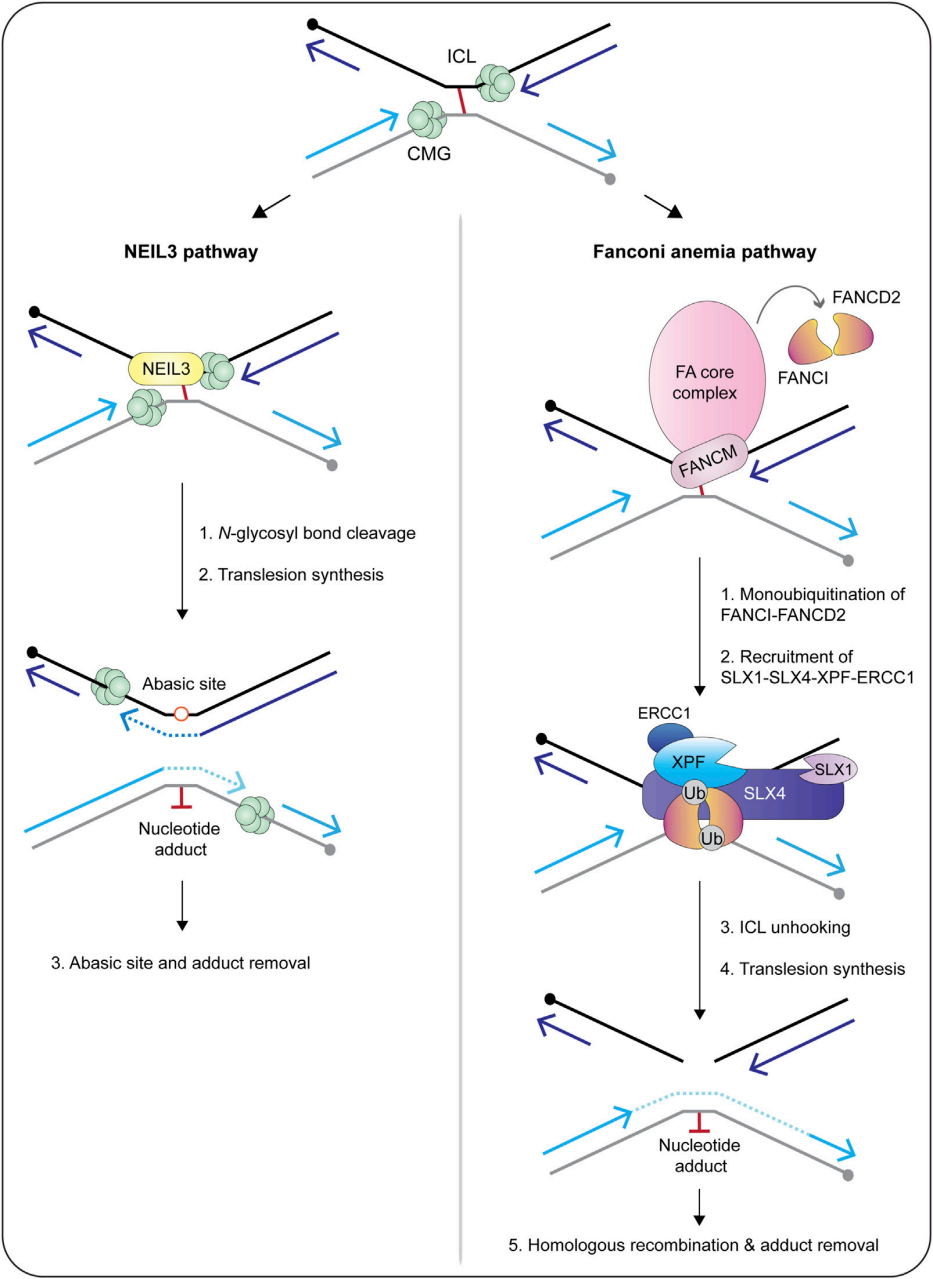
Current models of replication-coupled ICL repair by the NEIL3 glycosylase **(left)** and Fanconi anemia **(right)** pathways. Repair is activated when replisomes converge at the ICL. The NEIL3 pathway is preferentially activated by abasic and psoralen-induced crosslinks, whereas the Fanconi anemia pathway is activated by lesions caused by cisplatin, mitomycin C and acetaldehyde. In the NEIL3 pathway, one of the two *N*-glycosyl bonds that form the ICL are cleaved by the NEIL3 glycosylase, unhooking the crosslink without generating a double-strand break. This reaction does not require CMG unloading or FANCI-FANCD2. After unhooking, the gaps are filled in via translesion synthesis polymerases. If the NEIL3 pathway fails or the replisomes converge at a cisplatin-induced crosslink, the Fanconi anemia pathway is activated and repairs the lesion via dual incisions in the phosphodiester backbone. This reaction requires unloading of the CMG helicase and activation of the Fanconi anemia core complex. The core complex catalyzes the monoubiquitination of FANCI-FANCD2, causing it to clamp around double-stranded DNA. This is thought to trigger the recruitment of SLX4 and its associated endonucleases to execute crosslink unhooking via dual incisions in the phosphodiester backbone. The resulting double-strand break is repaired through the actions of translesion synthesis polymerases and homologous recombination. In both pathways, the nucleotide adduct is removed by nucleotide excision repair. Circles indicate 5′-termini. Arrowheads on nascent DNA indicate 3′-termini. Abbreviations: ICL, interstrand crosslink; Ub, ubiquitin.

Biochemical fractionation and coimmunoprecipitation experiments revealed that human SLX1-SLX4 is constitutively associated with XPF-ERCC1 throughout the cell cycle, forming the SX complex (Figure 6) (Wyatt et al., 2017; Panichnantakul et al., 2021). The SX complex is held together through direct contacts between the SLX4 MLR domain and the XPF N-terminal helicase-like domain (Fekairi et al., 2009; Svendsen et al., 2009; Kim et al., 2013; Guervilly et al., 2015; Hashimoto et al., 2015; Zhang et al., 2019). The interaction between SLX4 and XPF is critical for the repair of DNA interstrand crosslinks and a subset of Fanconi anemia patients harbor mutations in *SLX4/FANCP* (Kim et al., 2011; Stoepker et al., 2011; Schuster et al., 2013) or *XPF/FANCQ* (Bogliolo et al., 2013). Interstrand crosslinks are exceptionally toxic lesions that covalently link both strands of the double helix and prevent strand separation, which blocks DNA transcription and replication. These lesions result from reactive aldehydes produced during specific metabolic processes (e.g., alcohol metabolism), lipid metabolism, or exposure to chemotherapeutics (e.g., psoralen, cisplatin and mitomycin C). The chemistry and three-dimensional structure of an interstrand crosslink in duplex DNA influences downstream repair (for a review, see Housh et al., 2021). For example, the NEIL3 glycosylase is critical for the repair of abasic and psoralen-induced crosslinks, whereas the Fanconi anemia pathway repairs lesions caused by acetaldehyde, cisplatin and mitomycin C (Figure 7) (Semlow et al., 2016; Hodskinson et al., 2020; Li et al., 2020). The reader is referred to Semlow and Walter (2021) for an in-depth review of vertebrate interstrand crosslink repair.

The majority of interstrand crosslink repair occurs during S-phase, which is the phase of the cell cycle when the SX complex predominates (Figure 6) (Wyatt et al., 2017; Panichnantakul et al., 2021). In a cell-free system using *Xenopus* egg extracts, the interstrand crosslink is recognized when two replication forks converge (Figure 7) (Raschle et al., 2008; Zhang et al., 2015). Under certain conditions, the replication fork can collapse, which triggers the recruitment of the Fanconi anemia core complex (Figure 7) (Knipscheer et al., 2009; Wu et al., 2019). The core complex monoubiquitinates the FANCI-FANCD2 heterodimer, which facilitates the recruitment and coordination of DNA endonucleases (e.g., SLX1-SLX4, XPF-ERCC1), translesion synthesis polymerases (e.g., Rev1, pol ζ) and homologous recombination proteins (e.g., RAD51) (Meetei et al., 2003; Sims et al., 2007; Smogorzewska et al., 2007; Knipscheer et al., 2009; Ho et al., 2011; Long et al., 2011; Yamamoto et al., 2011; Klein Douwel et al., 2014; Budzowska et al., 2015; Rickman et al., 2015; Klein Douwel et al., 2017; Bezalel-Buch et al., 2020). Crosslink “unhooking” is a critical step of DNA interstrand crosslink repair (Figure 7). This reaction uses nucleases to introduce nicks on the 5′- and 3′-sides of the crosslink. After unhooking, one strand of the helix contains an adduct and a single-stranded gap that is filled in by error-prone translesion synthesis (Roy and Scharer, 2016). The other strand contains a double-strand break that is repaired through homologous recombination (Figure 7) (Michl et al., 2016).

The SLX4 scaffold has a critical role in crosslink unhooking, functioning to recruit XPF-ERCC1 to the lesion (Klein Douwel et al., 2014; Klein Douwel et al., 2017). The SLX4 UBZ domains are needed to recruit SLX4 to interstrand crosslinks (Yamamoto et al., 2011), as is the loading of FANCI-FANCD2 (Klein Douwel et al., 2014). Remarkably, biochemical experiments revealed that the SLX4-XPF-ERCC1 complex is sufficient for crosslink unhooking (Hodskinson et al., 2014; Klein Douwel et al., 2014; Klein Douwel et al., 2017; Hoogenboom et al., 2019). First, an N-terminal fragment of murine SLX4, spanning residues 1–758, was sufficient to stimulate crosslink unhooking by XPF-ERCC1 *in vitro* (Hodskinson et al., 2014). These experiments monitored the incision of DNA substrates containing mildly distorting lesions (i.e., nitrogen mustard). Second, the depletion of SLX4 or XPF from *Xenopus* egg extracts inhibited the unhooking and repair of cisplatin-induced interstrand crosslinks (Klein Douwel et al., 2014; Klein Douwel et al., 2017). Together, these studies provide strong biochemical evidence that SLX1 is dispensable for interstrand crosslink unhooking. Nevertheless, given the intricacies of interstrand crosslink repair, it will be important to determine whether SLX1-SLX4 and XPF-ERCC1 collaborate to unhook certain types of DNA interstrand crosslinks. Another possibility is that SLX1 resolves recombination intermediates that occur in the late stages of interstrand crosslink repair.

In addition to its role in interstrand crosslink repair, the SX complex may also be involved in processing 3′-ends in APE2-deficient cells. APE2 is an apurinic/apyrimidinic (AP) endonuclease that displays robust 3′-phosphodiesterase and 3′-5′ exonuclease activities but weak AP endonuclease activity (Hadi and Wilson, 2000; Burkovics et al., 2006). In human cells, APE2 has a critical role in reversing blocked 3′-ends to generate the free 3′-hydroxyl needed for DNA synthesis during homologous recombination (Mengwasser et al., 2019; Alvarez-Quilon et al., 2020). Interestingly, SLX4, XPF and ERCC1 are required for the viability of APE2-deficient cells (Alvarez-Quilon et al., 2020). This points towards a role for SX in processing unextendible recombination intermediates and cleaving near the branchpoint to remove the 3′-block. Further studies are needed to explore this intriguing model.

### 5.2 The SMX Tri-Nuclease Complex

When human cells enter prometaphase, MUS81-EME1 is recruited to the SX complex, leading to the formation of a tri-nuclease complex called SMX (Figure 6) (Wyatt et al., 2013; Wyatt et al., 2017). The mechanism of SMX assembly involves direct interactions between the SLX4 SAP domain and the MUS81 N-terminal HhH domain (Fekairi et al., 2009; Nair et al., 2014, our unpublished data). Furthermore, SMX assembly requires phosphorylation of the SLX4 SAP and MUS81 N-HhH domains in early mitosis, events that are driven by the CDK1 and CK2 kinases, respectively (Duda et al., 2016; Palma et al., 2018). *In silico* experiments identified six potential CDK1 target sites in and around the SLX4 SAP domain (Duda et al., 2016). SLX4 mutants harboring alanine substitutions of these six residues failed to coimmunoprecipitate MUS81 (Duda et al., 2016). On the other hand, CK2 phosphorylated one residue in the MUS81 N-HhH domain, Ser87, and alanine substitution reduced the ability of MUS81 to coimmunoprecipitate SLX4 (Palma et al., 2018). Interestingly, phosphorylation of S87 increased in response to replication stress and in cells transitioning from G2 to prophase; phosphorylation of S87 disappeared as soon as cells entered metaphase (Palma et al., 2018).

Recombinant SMX is a promiscuous structure-selective endonuclease that cleaves a broad range of DNA secondary structures, including replication fork structures and recombination intermediates (Wyatt et al., 2017). The observation that SMX cleaves a range of branched DNA structures with high catalytic efficiency reveals an interesting dichotomy. On the one hand, it provides biochemical evidence for the roles of SMX in DNA repair and genome stability, which include: fragile site cleavage and mitotic DNA synthesis (Naim et al., 2013; Ying et al., 2013; Guervilly et al., 2015; Minocherhomji et al., 2015; Garribba et al., 2020), the resolution of recombination intermediates (Wechsler et al., 2011; Castor et al., 2013; Garner et al., 2013; Wyatt et al., 2013; Nair et al., 2014; Sarbajna et al., 2014) and telomere homeostasis (Wan et al., 2013; Wilson et al., 2013; Ouyang et al., 2015; Sarkar et al., 2015; Panier et al., 2019; Verma et al., 2019; Yang et al., 2020). On the other hand, the promiscuous nuclease activity of SMX makes it a potential threat to chromosome integrity, particularly during S-phase. This hints at the existence of one or more regulatory mechanisms to control SMX *in vivo*. Indeed, the temporally regulated assembly of SMX is critical for genome stability, and premature assembly leads to catastrophic DNA damage and chromosome pulverization. In these experiments, SMX was forced to assemble in S-phase because of constitutive CDK1 activity, which was achieved through chemical inhibition of the inhibitory phosphatase WEE1 (Duda et al., 2016) or genetic manipulation of *Cdk1* (Szmyd et al., 2019). In another approach, the expression of a phosphomimetic MUS81 S87D mutant induced extensive DNA damage in S-phase, as well as pulverized chromosomes (Palma et al., 2018). The DNA damage was largely dependent on the nuclease activity of MUS81, consistent with the biochemical data showing that within the SMX complex, MUS81-EME1 is the predominant nuclease that cleaves replication fork structures (Duda et al., 2016; Wyatt et al., 2017; Palma et al., 2018; Szmyd et al., 2019). The observation that SLX4 relaxes the substrate specificity of MUS81-EME1 bears some resemblance to the promiscuous nuclease activity exhibited by SLX1-SLX4. Recent work has provided insight into the replication structures that could be targeted by the SMX complex in human cells. Specifically, van Wietmarschen et al. showed that SLX4 and MUS81-EME1 are involved in the cleavage of DNA secondary structures in expanded TA-dinucleotide repeats, resulting in double-strand breaks and chromosome shattering (Van Wietmarschen et al., 2020). Further work showed that the double-strand breaks resulted from the cleavage of replication forks stalled in expanded (TA)_*n*_ repeats (Van Wietmarschen et al., 2020). However, chromosome shattering was only observed in cancer cells that lacked the WRN helicase, a member of the RecQ family that melts DNA secondary structures, and exhibited microsatellite instability, which refers to the genetic hypermutability that results from impaired DNA mismatch repair (Van Wietmarschen et al., 2020). This indicates that stalled replication forks are normally rescued by the WRN helicase. In the absence of WRN, SLX4 and MUS81-EME1 (presumably within the SMX complex), cleave the stalled replication forks to trigger replication fork restart. However, the frequency of replication fork cleavage is so high that the result is chromosome fragmentation and cell death (Van Wietmarschen et al., 2020). Further work is needed to determine whether this phenotype results from the SMX complex *per se* and if so, to determine if the mechanism of SMX assembly is deregulated (i.e., S-phase instead of mitosis) in specific cellular contexts.

Altogether, the picture emerges that SMX represents a versatile nuclease toolkit to remove a plethora of branched DNA structures that could compromise accurate DNA replication or chromosome segregation. One crucial challenge for the future is determining the abundance of SX and SMX at different cell cycle stages. For example, although we assume that all of the SLX1-SLX4 and MUS81-EME1 in early mitotic cells exists within the context of SMX, this has not been evaluated scientifically. It will be equally important to develop cell models that allow researchers to distinguish between SX- and SMX-dependent functions.

## 6 Macromolecular SLX4-Complexes: New Players on the Block

As discussed above, SLX4 provides the scaffold for assembling two macromolecular nuclease complexes (Figure 6). Increasing evidence points towards the presence of additional subunits that associate with SLX4. Remodelling of SX and SMX complexes through different combinations of interacting subunits could serve to uniquely modulate enzymatic activity or subcellular localization. For example, SLX4 interacts with a DNA helicase called RTEL1 (Figure 5A) (Takedachi et al., 2020). RTEL1 is a DNA helicase that has important roles in DNA replication, particularly at telomeres, functioning to unwind DNA secondary structures like R-loops and G-quadruplexes (Lansdorp and Van Wietmarschen, 2019). The SLX4-RTEL1 interaction promotes DNA replication by circumventing transcription-mediated obstacles, such as collisions between the replisome and the transcription machinery, in part by promoting the accumulation of FANCD2 near active RNA polymerase II (Takedachi et al., 2020). Remarkably, SLX4 promotes replication fork progression independently of its associated nucleases. Future work is needed to determine whether RTEL1 inhibits SLX4-associated nucleases or whether cells contain a novel subcomplex composed of SLX4 and RTEL1.

Recent work has also shed light on the interaction between SLX4 and the MSH2-MSH3 heterodimer (Figure 5A), which has important roles in mismatch repair and homologous recombination (Svendsen et al., 2009; Gonzalez-Prieto et al., 2015; Zhang et al., 2019; Young et al., 2020). Coimmunoprecipitation experiments showed that the interaction between SLX1-SLX4 and MSH2-MSH3 is consistent throughout the cell cycle, suggesting that MSH2-MSH3 may represent an integral component of the SX and SMX complexes (Young et al., 2020). Indeed, MSH2-MSH3 stimulated the ability of SLX1-SLX4 and SMX to cleave Holliday junctions and trinucleotide repeat loops, expanding the repertoire of DNA substrates that are cleaved by SLX4-nuclease complexes (Young et al., 2020). The interplay between SLX4 and MSH2-MSH3 was reviewed recently (Young and West, 2021).

SLX4IP is another protein that seems to represent a constitutive binding partner of SLX1-SLX4. As discussed below, SLX4IP has multifaceted roles in genome stability, including telomere homeostasis, cellular signaling pathways and DNA interstrand crosslink repair.

### 6.1 SLX4IP

#### 6.1.1 Discovery and Properties of SLX4IP

**FIGURE 8:**
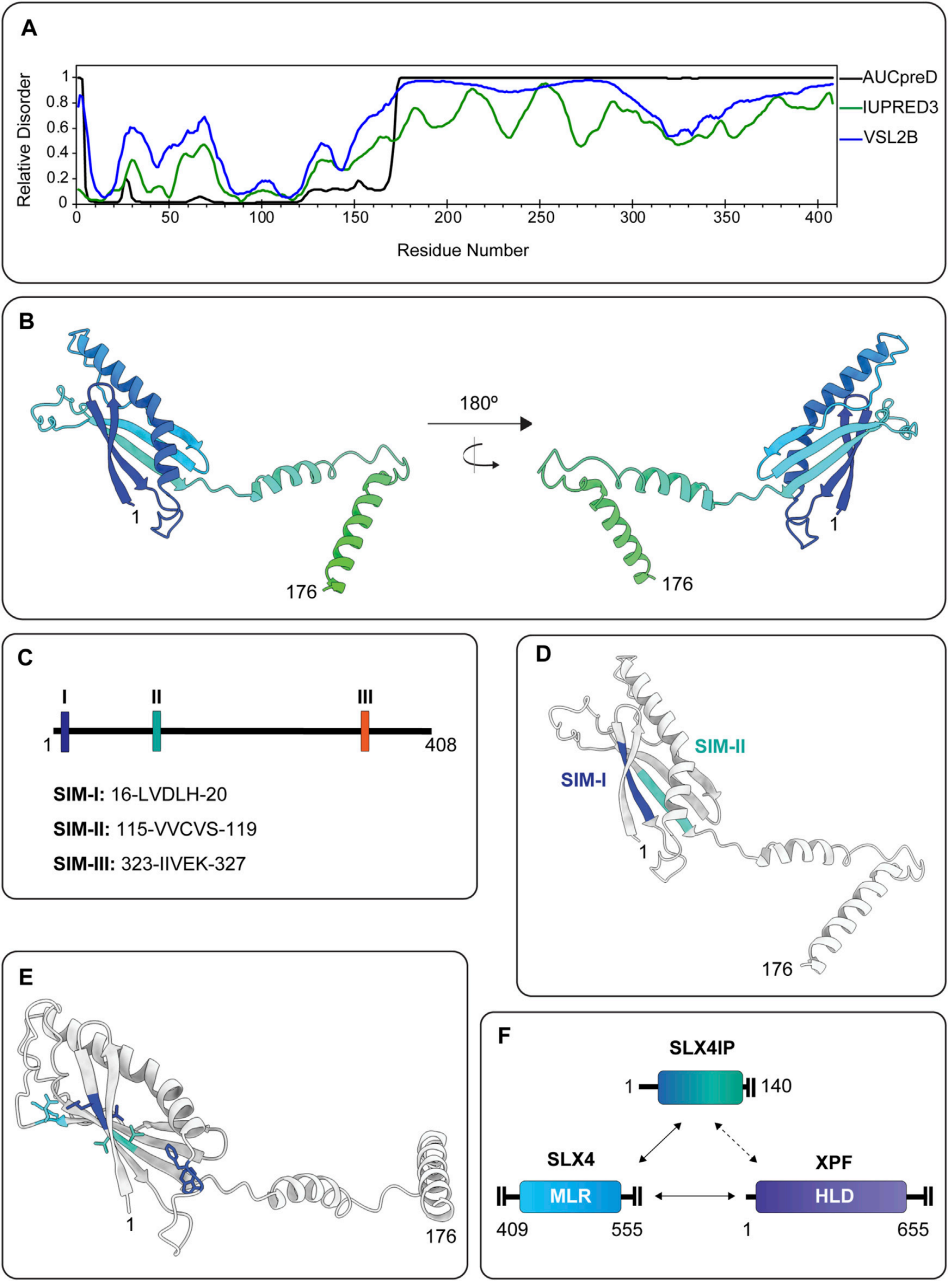
**(A)** Disorder probability plots for human SLX4IP using the AUCpreD (black trace) (Wang et al., 2016), IUPRED3 (green trace) (Erdos et al., 2021) and VSL2B (blue trace) (Peng et al., 2006) predictors. The N-terminus of SLX4IP is predicted to be relatively ordered whereas the C-terminus is predicted to be largely disordered, as indicated by a disorder tendency score >0.5. **(B)** Predicted structural architecture of the SLX4IP N-terminus (residues 1–176) shown in rainbow color and generated by AlphaFold (Jumper et al., 2021). **(C)** Linear representation of SLX4IP showing the approximate locations of three putative SIMs, designated as SIM-I, SIM-II and SIM-III and predicted using GPS-SUMO (Ren et al., 2009; Zhao et al., 2014). The amino acid sequence of each motif is shown below the schematic. Numbers represent the first and last residue in the motifs. **(D)** Predicted structure of the SLX4IP N-terminus (residues 1–176), as generated by AlphaFold (Jumper et al., 2021). SIM-I and SIM-II are shown in color (blue and teal, respectively). **(E)** Predicted structure of the SLX4IP N-terminus (residues 1–176), as generated by AlphaFold (Jumper et al., 2021). Residues that are necessary for the interaction with SLX4 are shown in stick format and colored as follows: L16/V17 (blue), W32/F33 (blue), L91/R92 (teal) and V115/V116 (light blue). **(F)** Graphical summary of the interactions that have been observed between the SLX4IP N-terminus, the SLX4 MLR domain and the XPF HLD (amino acid boundaries are indicated). Solid lines denote direct protein-protein interactions, whereas the dashed line represents an ambiguous interaction (i.e., current data does not conclusively demonstrate direct or indirect protein-protein interaction). Abbreviations: SIM, SUMO-interacting motif; MLR, MUS312-MEI9 interaction-like region; HLD, SF2 helicase-like domain.

SLX4IP was identified by mass spectrometry as an uncharacterized protein, C20orf94, that coimmunoprecipitated with SLX1 and SLX4 from human embryonic kidney extracts (Svendsen et al., 2009). *SLX4IP* is a vertebrate-specific gene that encodes a protein without recognizable domains (Svendsen et al., 2009). Human *SLX4IP* is located on the short arm of chromosome 20 and encodes a 408 amino acid protein that belongs to the uncharacterized protein family UPF0492. One large knowledge gap in our understanding of SLX4IP concerns its structure. Protein disorder predictions show that human SLX4IP contains a relatively well-ordered N-terminus and a highly disordered C-terminus (Figure 8A) (Xue et al., 2010; Meszaros et al., 2018; Hanson et al., 2019; Erdos et al., 2021; Robinson et al., 2021). Structural predictions suggest that the N-terminus is likely composed of α-helices and anti-parallel β-sheets (Figure 8B), whereas the structure of the C-terminus cannot be predicted accurately (Jumper et al., 2021; Robinson et al., 2021). Further inspection of the SLX4IP sequence using GPS-SUMO reveals three putative SIMs (Ren et al., 2009; Zhao et al., 2014). SIM-I and SIM-II are predicted to form β-strands in the N-terminus, whereas SIM-III is in the disordered C-terminus (Figures 8C,D) (Panier et al., 2019; Zhang et al., 2019). Moving forward, it will be essential to determine whether these are *bona fide* SIMs (i.e., interact non-covalently with SUMO or SUMOylated proteins) and, if so, to characterize the binding preference for SUMO isoforms (i.e., SUMO-1/2/3) and identify the SUMOylated binding partner(s). The field will also benefit significantly from the three-dimensional structures of SLX4IP, although the highly disordered C-terminus may pose a significant challenge for traditional methods like X-ray crystallography.

#### 6.1.2 Structural Anatomy of SLX4IP Protein Complexes

Our understanding of the structural anatomy of the SLX1-SLX4-SLX4IP complex comes from a comprehensive set of molecular biology experiments. First, Svendsen et al. (2009) captured physical interactions between SLX4 and SLX4IP in a yeast two-hybrid assay, suggesting that these proteins likely contact each other directly. Of note, because this study did not test for physical interactions between SLX1 and SLX4IP, we cannot exclude a model in which SLX1 forms part of the binding interface. Domain mapping experiments that explored interactions between full-length SLX4 and SLX4IP deletion constructs, or *vice versa*, support the general conclusion that SLX4 and SLX4IP interact directly (Svendsen et al., 2009; Panier et al., 2019; Zhang et al., 2019). The interaction between SLX1-SLX4 and SLX4IP has since been investigated by additional laboratories and appears to be consistent throughout the cell cycle (Panier et al., 2019; Zhang et al., 2019). Below, we discuss our understanding of the region(s) of SLX4IP and SLX4 that mediate this protein-protein interaction.

The SLX4IP N-terminus contains two putative SIM domains (SIM I and II, Figures 8C–E), which are necessary for the interaction with SLX4. SLX4IP mutants harboring non-conservative mutations in either SIM-I (i.e., L16K/V17K) or SIM-II (i.e., V115K/V116K) did not coimmunoprecipitate or colocalize with SLX4 (Panier et al., 2019; Zhang et al., 2019). It will be important to determine if the L16K/V17K and V115K/V116K mutations impact the structure of SLX4IP. For example, it would be helpful to know whether the native aliphatic sidechains or predicted β-strands (Figure 8E) facilitate SLX4-binding and whether basic residues disrupt the predicted secondary structure. Another question is the role(s) of SLX4IP W32, F33, L91 and R92, which are located outside of the SIM domains (Figure 8E). Specifically, pairs of alanine substitutions (i.e., W32A/F33A or L91A/R92A) disrupt the ability of SLX4IP to coimmunoprecipitate or colocalize with SLX4 (Panier et al., 2019; Zhang et al., 2019). Altogether, these studies reveal several amino acids in the SLX4IP N-terminus that mediate the interaction with SLX4.

On the other hand, SLX4IP binds predominately to the SLX4 MLR domain (Figure 8F) (Panier et al., 2019; Zhang et al., 2019). Alanine substitutions of SLX4 residues L530, F545, Y546 and L550 significantly reduce the interaction with SLX4IP (Zhang et al., 2019). This is a perplexing observation because these residues also form the binding site for XPF (Hashimoto et al., 2015), raising the possibility of mutually exclusive interactions between SLX4 and SLX4IP or XPF-ERCC1. Alternatively, within the context of an SLX4 dimer (Yin et al., 2016), perhaps SLX4IP binds to the MLR domain of one protomer while XPF-ERCC1 engages the other. Both models assume that SLX4 contains one binding site for SLX4IP and XPF-ERCC1. However, because of the predicted prevalence of intrinsically disordered regions in SLX4 (Figure 3A), we should also consider conformational selection, avidity and fuzziness (Vogt et al., 2014; Olsen et al., 2017). Indeed, Panier et al. (2019) observed a modest interaction between SLX4IP and the SLX4 N-terminus (residues 1–200), which could underpin the residual interaction between SLX4IP and the SLX4 L530A/F545A/Y546A/L550Y mutant (Zhang et al., 2019). Together, these results could indicate that SLX4 contains two binding sites for SLX4IP: perhaps the MLR domain contains a higher affinity binding site, and the N-terminus contains a lower affinity binding site. One important goal for the future is to elucidate the binding mechanism between SLX4 and SLX4IP (e.g., stoichiometry and affinity). It would also be interesting to know whether SLX4IP-binding influences local structural changes to the SLX4 MLR domain and the conformational landscape that accompanies this process.

Another layer of complexity comes from the observation that SLX4IP interacts with XPF, a well-known and direct binding partner of SLX4 (Andersen et al., 2009; Fekairi et al., 2009; Munoz et al., 2009; Svendsen et al., 2009; Wan et al., 2013; Hashimoto et al., 2015). SLX4IP seems to use the same residues to bind XPF and SLX4 (Panier et al., 2019; Zhang et al., 2019). Likewise, XPF mutants that do not coimmunoprecipitate SLX4IP also show reduced interaction with SLX4 (Zhang et al., 2019). The inability to separate the interactions between SLX4, SLX4IP and XPF provides strong evidence that these proteins exist in a macromolecular complex (Figure 8F). Additional evidence comes from the observation that the stability of SLX4IP is compromised by the loss of either SLX4 or XPF (Panier et al., 2019; Zhang et al., 2019). Nevertheless, SLX4IP coimmunoprecipitates comparable amounts of XPF-ERCC1 from wild-type and SLX4-depleted human cells (Panier et al., 2019; Zhang et al., 2019). This observation implies that SLX4IP can reside in different subcomplexes, including an SLX1-SLX4-SLX4IP complex that contains additional SLX4-binding partners (e.g., XPF-ERCC1), as well as an SLX4IP-XPF-ERCC1 complex that might have unique functions in DNA repair or homologous recombination. One possibility is that the SLX4IP-XPF-ERCC1 complex has a critical role in cells that lack the SLX4 scaffold. For example, it would be interesting to determine whether Fanconi anemia patient cells that lack SLX4 (Kim et al., 2011; Stoepker et al., 2011) are abnormally reliant on the SLX4IP-XPF-ERCC1 complex for genome stability.

#### 6.1.3 Biological Roles of SLX4IP

There is considerable interest in understanding the biological roles of SLX4IP, and valuable insights may come from studying patients, tissues and cells that have altered expression or mutations of *SLX4IP.* For example, SLX4IP is over-expressed in Merkel cell carcinoma, a rare but aggressive type of non-melanoma skin cancer (Kotowski et al., 2019). Conversely, monoallelic deletions encompassing the first two exons of *SLX4IP* occur in approx. 30% of childhood acute lymphoblastic leukemia patients (Meissner et al., 2014). The nonsense-mediated mRNA decay pathway likely eliminates the mutant transcripts, although this has not been tested experimentally. *SLX4IP* expression is downregulated in aneuploid acute myeloid leukemia cells (Simonetti et al., 2019) and a subset of osteosarcoma cells that use the ALT pathway (Panier et al., 2019). Molecular studies have convincingly demonstrated that SLX4IP colocalizes with telomeres in ALT-positive cancer cells but not in telomerase-positive cells (Dejardin and Kingston, 2009; Panier et al., 2019; Robinson et al., 2020; Robinson et al., 2021). SLX4IP is likely recruited to telomeres through its interaction with SLX4, which in turn is directed to telomeric chromatin through its interaction with TRF2 (Wan et al., 2013) and other SUMOylated proteins (Ouyang et al., 2015).

While it is clear that SLX4IP resides at the telomeres in ALT cells, what remains controversial is the precise role that SLX4IP plays in ALT. Panier et al. showed that the loss of SLX4IP in U2OS osteosarcoma cells triggered an increase in several markers of ALT, including the formation of ALT-associated promyelocytic leukaemia bodies (APBs), telomeric DNA damage, telomere length heterogeneity, extrachromosomal c-circles and telomeric sister chromatid exchanges (Panier et al., 2019). These phenotypes point towards a role for SLX4IP in dampening ALT activity. However, this result has been debated as Robinson et al. showed that the loss of SLX4IP in U2OS or murine breast cancer D2.OR cells decreased the frequency of ALT markers, suggesting that SLX4IP stimulates ALT (Robinson et al., 2020). Additional experiments are needed to reconcile the apparent discrepancy between these two datasets. One potential confounding variable is the genetic heterogeneity that can occur between different laboratory cell strains. Nevertheless, both groups found that the loss of SLX4IP triggered telomere shortening, which indicates that SLX4IP is needed for productive ALT (i.e., telomere length maintenance) (Panier et al., 2019; Robinson et al., 2020).

**FIGURE 9:**
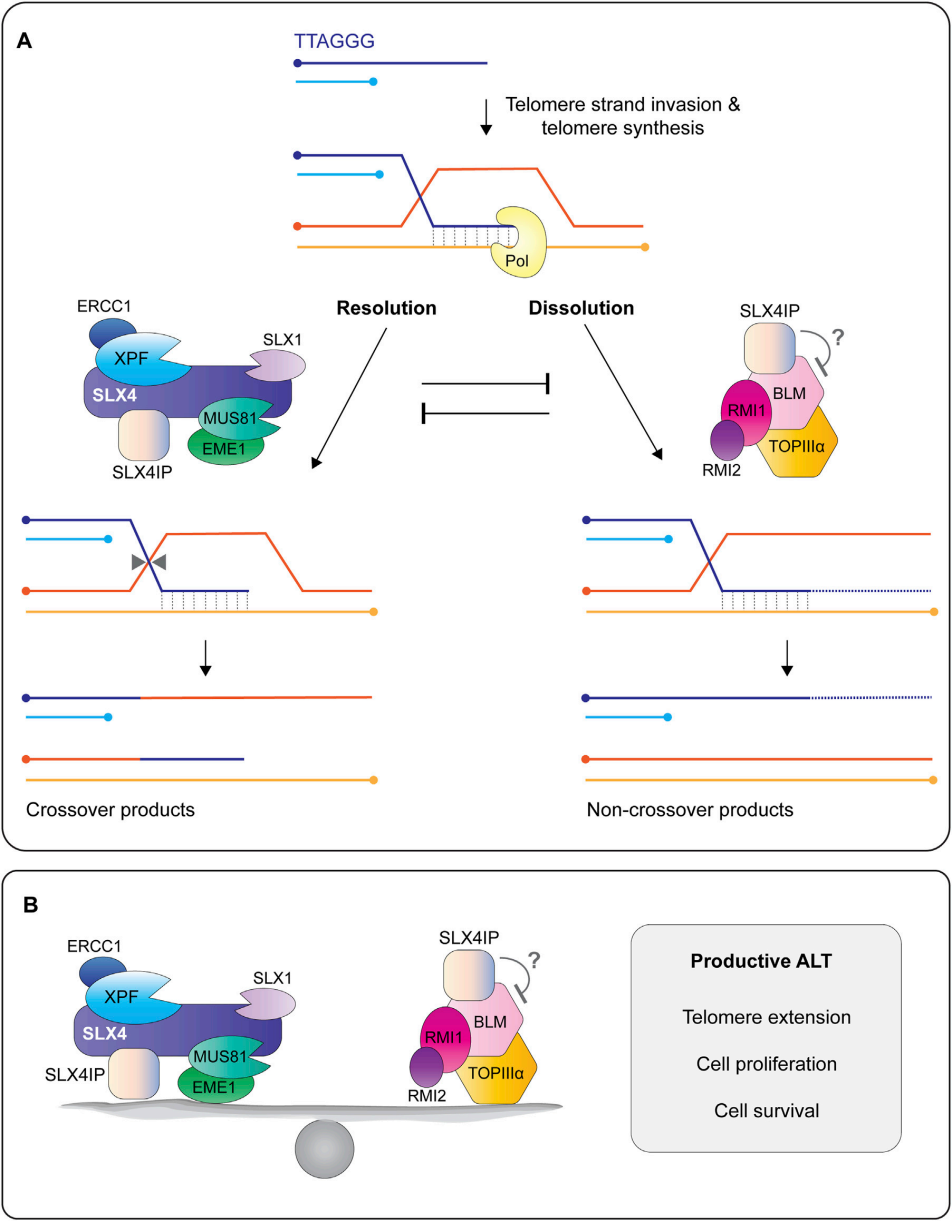
SLX4IP is a critical regulator of the alternative lengthening of telomeres (ALT) pathway. **(A)** The 3′ G-rich tail undergoes intertelomeric strand invasion, generating a branched DNA structure called a displacement-loop (D-loop). Strand invasion promotes break-induced telomere synthesis using one of several different polymerases, shown here as Pol. Telomere extension is regulated by the opposing actions of structure-selective endonucleases and a helicase-topoisomerase complex. In the resolution pathway **(left)**, the D-loop is cleaved/resolved by the endonucleases bound to SLX4, resulting a telomere exchange event (crossover) in the absence of telomere extension. In the dissolution pathway **(right)**, a protein complex containing the BLM helicase, TOPIIIα topoisomerase, and RMI1/2 proteins promotes branch migration and long tract telomere synthesis, followed by D-loop dissolution into non-crossover products. Recent work shows that SLX4IP functions in both pathways through its interactions with SLX4, XPF and BLM. Circles indicate 5′-termini. **(B)** Current model depicting SLX4IP as a protein that balances the two molecular pathways that regulate telomere length in ALT cells: resolution of recombining telomeres by SLX4 and its associated endonucleases and dissolution of recombining telomeres by the BTR complex. The presence of SLX4IP is essential for telomere length maintenance in ALT cells, as well as cell proliferation and survival. More work is needed to determine the mechanism(s) by which SLX4IP achieves this balance.

Several approaches have provided insight into how SLX4IP regulates ALT. Early work revealed that SLX4IP balances the actions of endonucleases and helicases at recombining telomeres (Figures 9A,B) (Panier et al., 2019; Robinson et al., 2020). Panier et al. showed that the depletion of SLX4 in U2OS cells lacking SLX4IP exacerbated ALT phenotypes and telomere recombination (Panier et al., 2019). This result provided the first clue that SLX4IP may have an SLX4-independent role in ALT. Indeed, the co-depletion of the BLM helicase was sufficient to rescue the telomere phenotypes exhibited by SLX4IP-deficient cells (Panier et al., 2019). Further work showed that SLX4IP uses its SIM-I and SIM-II motifs to interact directly with BLM (Panier et al., 2019). Collectively, these findings position SLX4IP as a nexus between the two molecular pathways that dictate telomere lengths in ALT: dissolution via BLM and resolution via SLX4 with its associated endonucleases (Figures 9A,B). Although SLX4IP does not inhibit the helicase activity of BLM *in vitro* (Panier et al., 2019), it will be important to determine whether SLX4IP alters the enzymatic properties of the BTR complex and SLX1-SLX4 (in the presence and absence of XPF-ERCC1 and MUS81-EME1) towards ALT recombination intermediates.

More recently, SLX4IP has emerged as a critical regulator of the telomere proteome. Robinson et al. used quantitative proteomics of isolated chromatin to compare the composition of telomere segments from SLX4IP-proficient and deficient U2OS cells (Robinson et al., 2021). They observed that SLX4IP levels correlated with the SUMOylation of several proteins, most notably XPF and the shelterin protein RAP1. Further work on the link between SLX4IP and SUMOylation showed that SLX4IP facilitated the interaction between SLX4 and PIAS1 (Robinson et al., 2021), an E3 ligase that plays a central role as a transcriptional coregulator of numerous cellular pathways. SLX4IP also stimulated the biochemical reaction between PIAS1 and RAP1 *in vitro*, leading to the SUMOylation of human RAP1 K240 (Robinson et al., 2021).

**FIGURE 10:**
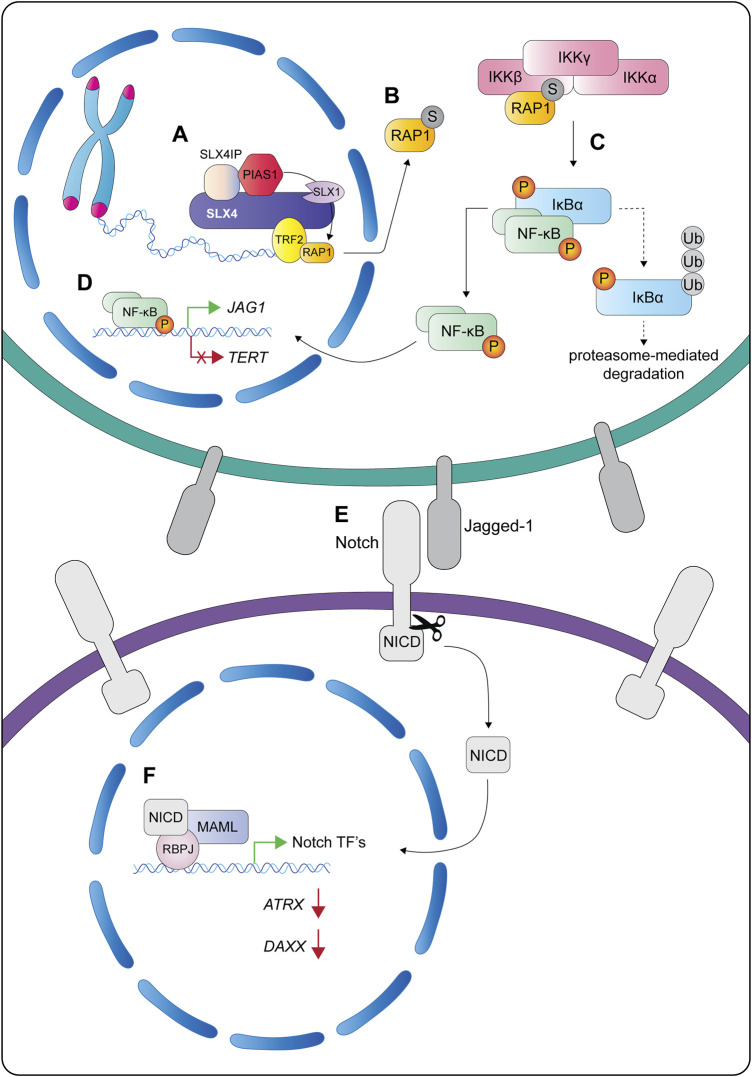
Proposed model for the role of SLX4IP in promoting RAP1 SUMOylation and activation of the IKK—NF-κB—Notch signaling axis in cells using the ALT pathway. **(A)** SLX4IP is recruited to ALT telomeres through its interaction with SLX4. The presence of SLX4IP stimulates PIAS1 to SUMOylate RAP1 on K240. The SUMOylation of RAP1 reduces its ability to interact with TRF2 and SLX4, thus promoting its redistribution from the nucleus into the cytoplasm **(B)**. There, SUMOylated RAP1 activates NF-κB signaling by binding to the IKKβ subunit of the IKK complex. **(C)** The interaction between SUMOylated RAP1 IKKβ triggers the phosphorylation and proteasome-dependent degradation of IκBα, thus liberating NF-κB and promoting its translocation into the nucleus. **(D)** Once in the nucleus, NF-κB represses the transcription of *TERT* (the catalytic subunit of telomerase), which is thought to sustain the ALT mechanism. Additionally, NF-κB activates the transcription of several genes, including *JAG1*, which encodes a cell surface protein and Notch ligand called Jagged-1. **(E)** The binding of Jagged-1 to a Notch receptor on a neighboring cell initiates Notch signaling by releasing the NICD through a cascade of proteolytic cleavages (depicted by scissors). The NICD is a transcription factor that works together with additional coactivators (RBPJ and MAML) to activate transcription of target genes, some of which include Notch transcription factors **(F)**. This is thought to establish a feed-forward loop that sustains the ALT phenotype by controlling the expression of ALT-associated genes (e.g., *ATRX*, *DAXX*). Abbreviations: S, SUMO; P, phosphorylation; Ub, ubiquitin; NICD, Notch intracellular domain; ALT, alternative lengthening of telomeres.

One particularly intriguing observation is that SUMOylated RAP1 accumulates in the cytosol, where it binds to the IKKβ subunit of the heterotrimeric IKK complex (Figure 10) (Teo et al., 2010; Robinson et al., 2021). This interaction triggers the phosphorylation and degradation of IκBα, thus liberating NF-κB and promoting its translocation into the nucleus (Figure 10). NF-κB is a transcription factor that is constitutively active in many different types of cancer and can accelerate cell proliferation, inhibit apoptosis, promote cell migration and invasion, and stimulate angiogenesis and metastasis (Taniguchi and Karin, 2018). For example, NF-κB induces the expression of Jagged-1, which in turn stimulates Notch signaling (Figure 10). Interestingly, Robinson et al. observed that ALT cells harbor a Notch-responsive gene signature that can be disrupted by depleting SLX4IP (Robinson et al., 2021). Some of the most notable targets that are repressed in ALT cells include *TERT* (the catalytic subunit of telomerase) and the histone chaperone subunits *ATRX* and *DAXX*. Inactivating the NF-κB or Notch pathways through *SLX4IP* gene disruption or RAP1 K240A mutation (i.e., SUMOylation defective) alleviates the repression of *TERT*, *ATRX* and *DAXX* (Robinson et al., 2021). Altogether, these data establish SLX4IP-mediated SUMOylation of RAP1 as a new modulator of the NF-κB signaling pathway (Figure 10). It remains to be determined if NF-κB or Notch signaling regulates the expression of other factors that help establish an ALT-permissive cellular environment. For example, the loss of SLX4IP in ALT cells upregulates *BLM* gene transcription and results in elevated levels of BLM protein (Panier et al., 2019). The accumulation of BLM could disrupt the balance between dissolution and resolution at recombining telomeres, which may help explain the telomere shortening seen in ALT cells lacking SLX4IP.

SLX4IP is also involved in interstrand crosslink repair in non-ALT cells. The disruption of *SLX4IP* in HEK293 cells caused hypersensitivity to mitomycin C, but not to ultraviolet radiation, ionizing radiation, or camptothecin (Zhang et al., 2019). Given that SLX4IP interacts with SLX4 and XPF, it will be important to elucidate the substrate specificity and enzymatic properties of SX complexes with and without SLX4IP. For example, SLX4IP may stabilize the interaction between SLX1-SLX4 and XPF-ERCC1 or facilitate substrate positioning in the XPF-ERCC1 active site. Both models are consistent with the observation that *SLX4IP* disruption caused a modest reduction in the efficiency of crosslink unhooking *in vivo* (Zhang et al., 2019). Another interesting question is whether SLX4IP modulates the nuclease activities of SX or SMX on downstream recombination intermediates (e.g., Holliday junction).

## 7 Discussion

This review describes how cells leverage and regulate the promiscuous Slx1/SLX1 nuclease to maintain genome stability. Through its interaction with the Slx4/SLX4 scaffold, Slx1/SLX1 has acquired the unique ability to cleave many types of branched DNA structures that form during DNA replication, repair and recombination. Our understanding of the mechanisms that underpin this atypical substrate specificity are coming into focus. Slx1 uses three distinct DNA-binding sites to ensure that DNA substrates are positioned accurately in the active site. DNA-bending has emerged as an important pre-requisite for substrate cleavage, which adds Slx1-Slx4 to the list of structure-selective endonucleases that use DNA-bending to facilitate branchpoint recognition and orientation in the active site. Together, these molecular safety pins ensure that Slx1-Slx4 cannot cleave linear stretches of ssDNA or dsDNA. Of note, this knowledge came from structural and biochemical studies of Slx1-Slx4^CCD^, which contains the minimal components for catalytic activity, leaving open the possibility that Slx4 may contribute to substrate recognition. Indeed, recent work reveals that the Slx4 SAP domain contributes to DNA-binding and cleavage. Structural and biochemical studies of full-length Slx1-Slx4/SLX1-SLX4 are clearly warranted, although we acknowledge that the lack of well-defined secondary structures in Slx4/SLX4 could make this a formidable challenge.

During the evolution of human SLX4, the protein acquired several domains and motifs that interact with functionally diverse proteins. Through these interactions, SLX4 has emerged as a central player in genome integrity. The functions of SLX4 in genome stability are best understood within the context of its associated structure-selective endonucleases: SLX1, MUS81-EME1 and XPF-ERCC1. SLX4 provides the hub for assembling these nucleases into at least two different macromolecular complexes: SX and SMX.

Molecular studies indicate that SX has critical functions in DNA interstrand crosslink repair and an emerging role in unblocking 3′-ends to facilitate DNA synthesis during homologous recombination. The role(s) fulfilled by SLX1 in these processes, if any, remains elusive. To that end, we eagerly await the reconstitution and biochemical analysis of full-length SX.

We have a better understanding of the SMX complex, which can be thought of as a molecular Swiss-army knife that removes branched DNA structures to ensure accurate DNA duplication and chromosome segregation. Interestingly, MUS81-EME1 is the predominant nuclease in SMX that cleaves replication intermediates, including those that are refractory to cleavage by MUS81-EME1 alone. More work is needed to understand how the activity of SLX1 is dampened within the SMX complex. In principle, the activity of SMX towards replication structures could have deleterious consequences during S-phase. However, cells have evolved regulatory mechanisms to control the activity of SMX. The best-characterized mechanism involves the temporally regulated assembly of SMX in prometaphase, at which time MUS81-EME1 is recruited to the SX complex. However, there are still several gaps in our knowledge of the mechanism that underpins SMX assembly. For example, how does phosphorylation promote the interaction between SLX1-SLX4 and MUS81-EME1? Reciprocally, does dephosphorylation trigger SMX disassembly?

In addition to the structure-selective endonucleases, SLX4 interacts with many other proteins that regulate genome stability (i.e., TRF2-RAP1, RTEL1, MSH2-MSH3 and SLX4IP). As such, the field is now faced with three challenges: i) determine whether cells contain a pool of “free” SLX1-SLX4 and if not, ii) identify constitutive binding partners of SLX1-SLX4 and iii) revisit the core functions ascribed to SLX1-SLX4 within the context of these new players. We also have a lot to learn about how these different macromolecular complexes are recruited to chromatin.

SLX4IP has emerged as a top candidate for an integral component of the SX and SMX complexes, making direct contacts with SLX4 and potentially XPF. Aside from three putative SIMs, SLX4IP lacks discernible structural and catalytic domains and may fulfill a scaffolding role within the SX or SMX complexes. Like SLX4, SLX4IP is consistently detected at the telomeres of ALT cells. Here, SLX4IP is needed to balance the opposing functions of structure-selective endonucleases and RecQ helicases, most notably BLM. Exactly how this is achieved remains elusive. In an unexpected twist, SLX4IP recently emerged as a key regulator of the telomere proteome and an SLX4-dependent effector of RAP1 SUMOylation. Surprisingly, SUMOylated RAP1 drives ALT by activating the NF-κB and Notch signaling pathways. Considerably more work is needed to dissect the structural anatomy of SLX4IP and the roles that it fulfills within ALT and non-ALT cells.

In closing, the advances that are being made in our understanding of the structure and multifaceted functions of macromolecular SLX4-complexes will no doubt feed into strategies for the molecular characterization of cancers and cancer-predisposing syndromes, the development of diagnostic, prognostic or predictive biomarkers, and the design of more efficacious cancer treatments.
